# Knowledge-primed neural networks enable biologically interpretable deep learning on single-cell sequencing data

**DOI:** 10.1186/s13059-020-02100-5

**Published:** 2020-08-03

**Authors:** Nikolaus Fortelny, Christoph Bock

**Affiliations:** 1 CeMM Research Center for Molecular Medicine of the Austrian Academy of Sciences, Vienna, Austria; 2 Department of Laboratory Medicine, Medical University of Vienna, Vienna, Austria

**Keywords:** Deep learning, Artificial neural networks, Single-cell sequencing, Gene regulation, Cell signaling networks, Functional genomics, Interpretable machine learning, Bioinformatic modeling

## Abstract

**Background:**

Deep learning has emerged as a versatile approach for predicting complex biological phenomena. However, its utility for biological discovery has so far been limited, given that generic deep neural networks provide little insight into the biological mechanisms that underlie a successful prediction. Here we demonstrate deep learning on biological networks, where every node has a molecular equivalent, such as a protein or gene, and every edge has a mechanistic interpretation, such as a regulatory interaction along a signaling pathway.

**Results:**

With knowledge-primed neural networks (KPNNs), we exploit the ability of deep learning algorithms to assign meaningful weights in multi-layered networks, resulting in a widely applicable approach for interpretable deep learning. We present a learning method that enhances the interpretability of trained KPNNs by stabilizing node weights in the presence of redundancy, enhancing the quantitative interpretability of node weights, and controlling for uneven connectivity in biological networks. We validate KPNNs on simulated data with known ground truth and demonstrate their practical use and utility in five biological applications with single-cell RNA-seq data for cancer and immune cells.

**Conclusions:**

We introduce KPNNs as a method that combines the predictive power of deep learning with the interpretability of biological networks. While demonstrated here on single-cell sequencing data, this method is broadly relevant to other research areas where prior domain knowledge can be represented as networks.

## Introduction

Deep learning using artificial neural networks (ANNs) has reached unprecedented prediction performance for complex tasks in multiple fields, including image recognition [1–3], speech recognition [4, 5], natural language processing [6–10], board and computer games [11–13], and autonomous driving [14, 15]. There is tremendous potential for deep learning in biology and medicine [16–19], as illustrated by initial applications in medical image classification [20], brain image segmentation [21], epigenome prediction [22], DNA/RNA binding analysis [23], and RNA splicing inference [24, 25]. Recently, deep learning has also shown promising results in the analysis of single-cell RNA sequencing (RNA-seq) datasets, facilitated by large single-cell datasets [26–30].

For certain specialized tasks with ample training data, ANNs already achieve prediction performance superior to that of human experts. However, the trained ANNs typically lack interpretability, i.e., the ability to provide human-understandable, high-level explanations of how they transform inputs (prediction attributes) into outputs (predicted class values). This lack of interpretability is a major limitation to the wider application of deep learning in biology and medicine—not only because it reduces trust and confidence in using such predictions for high-stakes applications such as clinical diagnostics [17, 18], but also because it misses important opportunities for data-driven biological discovery using deep learning.

Pioneering research aimed at making deep learning models interpretable and informative for biological applications focused primarily on ex post analysis of trained ANNs, for example by identifying inputs that result in specific predictions [23, 31, 32] or by analyzing the compressed layers of autoencoders [33]. A complementary approach is the ex ante engineering of deep learning architectures for built-in biological interpretability, for example by including domain knowledge from structural biology [34, 35], biophysical regulation of transcription [36, 37], gene annotations [38], cancer growth [39], genetic screens [40], or by combining knowledge from different domains [41–43].

Here we demonstrate the feasibility of deep learning on biological networks, including signaling pathways and gene-regulatory networks. We introduce knowledge-primed neural networks (KPNNs) as a broadly applicable framework for interpretable deep learning and biological discovery. We construct KPNNs from public annotation data for cellular signaling and transcription regulation, such that each node corresponds to a protein or a gene, and each edge corresponds to a regulatory relationship that has been documented in biological databases. We train the KPNNs based on single-cell RNA-seq data for the cell type or biological model of interest, using an optimized learning method that enhances the biological interpretability of the trained models.

Conceptually, we conceive cells as living “information processing units” [44, 45] that perform network-based “biological calculations” to regulate cell state [45–47]. KPNNs are designed to perform similar calculations in silico, predicting cell state from single-cell RNA-seq data in deep neural networks that are constructed based on biological knowledge. Because KPNNs capture key aspects of the cell’s regulatory machinery, we hypothesize that KPNN learning will give rise to interpretable models. To enable interpretability, we exploit three modifications to generic deep learning that enhance the interpretability of KPNNs: (i) repeated network training with random deletion of hidden nodes (a technique known as dropout [48]), which yields robust results in the presence of network redundancy; (ii) dropout on input data in order to enhance quantitative interpretability of node weights; and (iii) training on control inputs to normalize for the uneven connectivity of biological networks.

We validate KPNNs for interpretable deep learning using simulated data with known ground truth, we compare them to other machine learning algorithms including the ex post biological interpretation of feature weights, and we demonstrate the practical use of KPNNs in five biological applications with publicly available single-cell RNA-seq datasets: T cell receptor signaling [49], immune cells in the Human Cell Atlas [50], clinical subtypes of Langerhans cell histiocytosis [51], cancer cell development in acute myeloid leukemia [52], and cancer cell subtypes in glioblastoma [53]. Overall, our results establish KPNNs as a new form of interpretable deep learning that may be broadly useful for biological discovery based on large-scale datasets.

## Results

### KPNNs establish deep learning on biological networks

Deep learning using artificial neural networks (ANNs) seeks to “learn” (i.e., approximate in a generalizable way) the complex relationship between a set of prediction attributes (e.g., single-cell transcriptomes) and the corresponding class values (e.g., cell states). The learning process typically starts from a generic, fully connected, feedforward ANN—which is a directed acyclic graph organized in layers such that each node receives input from all nodes in the previous layer and sends its output to all nodes in the next layer. During the learning process, edge weights are randomly initiated and then updated iteratively based on training data, seeking to improve the accuracy with which the ANN transforms the inputs into corresponding class values. With enough training data, large multi-layer ANNs can learn highly complex relationships between prediction attributes and class values, without requiring any prior domain knowledge. However, the resulting trained ANNs lack interpretability, given that their nodes, edges, and weights do not correspond to meaningful domain concepts.

**Fig. 1:**
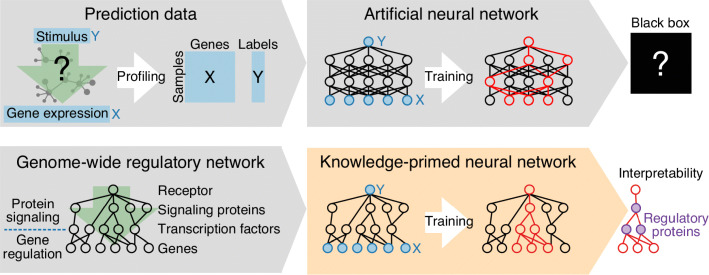
Interpretable deep learning with knowledge-primed neural networks (KPNNs). Deep learning provides a powerful method for predicting cell states from gene expression profiles. However, generic artificial neural networks (ANNs, top row) are “black boxes” that provide little insight into the biology that underlies a successful prediction – for two reasons: (i) hidden nodes and edges in an ANN have no biological equivalent, which makes it difficult assign a biological interpretation to the weights of a fitted ANN model, and (ii) ANNs are inherently instable, and very different networks can achieve similar prediction performance. Knowledge-primed neural networks (KPNNs, bottom row) enable interpretable deep learning on biological networks by exploiting structural analogies between biological networks (such as the signaling pathways and gene-regulatory networks that regulate cell state) and the feed-forward neural networks used for deep learning. In KPNNs, each network node corresponds to a protein or a gene, and each edge corresponds to a potential regulatory relationship that has been observed and annotated in public databases. Weights within the KPNN are obtained by a deep learning method that has been optimized for interpretability, and the learned weights are interpreted as estimates of the regulatory importance of the corresponding signaling protein or transcription factor

To overcome the lack of interpretability of deep learning on ANNs, we sought to embed relevant biological domain knowledge directly into the neural networks that are trained by deep learning (Fig. 1). To that end, we replaced the fully connected ANNs of generic deep learning with networks derived from prior knowledge of biological networks, thereby creating “knowledge-primed neural networks” (KPNNs). In KPNNs, each node corresponds to a protein or a gene, and each edge corresponds to a potential regulatory relationship that has previously been observed in any biological context and annotated in public databases. By integrating a broad range of previously documented regulatory relationships, we thus assume that most regulatory relationships relevant to the biological system of interest have already been observed in other contexts, and we use deep learning on KPNNs to contextualize this prior knowledge with single-cell RNA-seq training data for the investigated biological system.

We constructed KPNNs to reflect the typical flow of information in cells, where signals are transduced from receptors via signaling proteins to transcription factors, which in turn induce changes in gene expression. The expression levels of the regulated genes were used as input nodes, whose values can be measured by single-cell RNA-seq. Signaling proteins and transcription factors were modeled as hidden nodes, and we infer the corresponding node weights during the learning process. Finally, receptors constitute the output nodes of the KPNN, capturing phenotypic cell states and the ability of individual cells to interact with their environment.

KPNNs can be trained in much the same way as ANNs (see the “Methods” section for details), based on single-cell RNA-seq data for cells in different states (e.g., subtypes of cancer and immune cells) or under different environmental exposures (e.g., receptor-stimulated vs unstimulated cells). After successful completion of the learning process, each fitted edge weight is taken as an indicator for the relevance of the corresponding regulatory relationship in the investigated biological system. We then derive node weights from the edge weights, in order to identify signaling proteins and transcription factors that are likely relevant in this biological system. KPNNs thus exploit the ability of deep learning algorithms to assign meaningful weights across multiple hidden layers in KPNNs, thereby identifying and prioritizing relevant regulatory proteins for experimental validation and biological interpretation.

As the first biological test case for interpretable deep learning using KPNNs, we chose our recent single-cell RNA-seq dataset measuring cellular response to T cell receptor (TCR) stimulation in a standardized in vitro model [49]. The TCR signaling pathway, which orchestrates the transcriptional response to antigen detection in T cells, is well-suited for evaluating our method, given the pathway’s complexity and its well-characterized nature. To construct a dedicated TCR KPNN (Additional file 1: Fig. S1a), we connected the TCR (output node) to gene expression (input nodes) via shortest paths through a network of protein signaling and gene-regulatory interactions (hidden nodes). The resulting network was then reversed and trained to predict TCR stimulation from single-cell RNA-seq data: Gene expression (input nodes) provides the input for transcription factors (hidden nodes), whose outputs are used by signaling proteins (hidden nodes) to predict TCR stimulation (output node).

Deep learning on the TCR KPNN provided high prediction accuracy comparable to that of deep learning on ANNs (Additional file 1: Fig. S1b-c), despite the KPNN’s much lower number of edges (Additional file 1: Fig. S1d-f). Specifically, KPNNs predicted TCR stimulation with a median receiver operating characteristic (ROC) area under curve (AUC) value of 0.984 (interquartile range, 0.979 to 0.987), while ANNs with the same number of nodes (and many more edges) achieved a median ROC AUC value of 0.948 to 0.985 (interquartile range, 0.936 to 0.988 across all analyses; 0.938 to 0.989 for the best-performing number of network layers).

In summary, we have demonstrated that deep learning on KPNNs is practically feasible and that it can achieve comparable accuracies to deep learning on ANNs. These results hold the promise that deep learning on biological networks may yield trained network models that are both accurate and biologically interpretable.

### A biology-based network structure enables KPNN interpretability

To understand how the network structure of KPNNs may contribute to interpretable deep learning, we performed a systematic network analysis comparing the TCR KPNN with corresponding ANNs. As expected, the KPNN was much sparser than ANNs with the same number of nodes. For example, the best-performing ANN had 151-fold more edges than the KPNN (Additional file 1: Fig. S1d-f). However, the most striking differences between the KPNN and the corresponding ANNs referred to the structural properties of these networks.

**Fig. 2:**
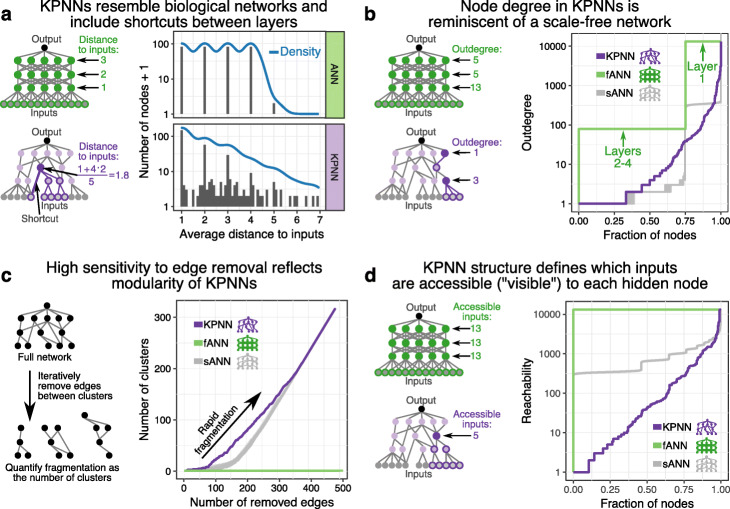
Comparative structural network analysis of KPNNs and ANNs. The TCR KPNN is compared to a fully connected ANN (fANN) with the same number of nodes and the same median depth as the KPNN, and to sparse ANNs (sANNs) where edges were randomly removed to match the edge number of the KPNN while retaining an intact network (results are shown for 50 random sANNs). **a** Average distance to the input nodes for all hidden nodes in the fANN (top) and KPNN (bottom). **b** Cumulative distribution of the outdegree of hidden nodes in the KPNN, the fANN, and averaged across the sANNs. **c** Assessment of network sensitivity to fragmentation upon removal of important edges in the KPNN, the fANN, and the sANNs. Edges were iteratively removed based on importance measured by their network betweenness value. **d** Cumulative distribution of reachability, which measures the number of input nodes each hidden node can connect to, shown separately for the KPNN, the fANN, and averaged across the sANNs

When we compared the KPNN to fully connected ANNs with the same number of nodes (fANN) and to sparse ANNs with the same number of edges as the KPNN (sANN, see the “Methods” section for details), the KPNN contained many deep connections (“shortcuts”), contrasting with the strictly layered network architecture of the ANNs (Fig. 2a, Additional file 1: Fig. S2a). Because each node in the fully connected ANNs is connected to all nodes of both the previous and the following layer, the distance from a given node to any node of the input layer is the same, depending only on the node’s layer. In contrast, distances in the KPNN differed widely due to the presence of shortcuts that connect certain parts of the network much more directly with the input layer.

The KPNN was further characterized by unique patterns of network connectedness. Most notably, the distribution of node outdegrees (the outdegree measures the number of edges that leave a node) resembled an exponential distribution for the KPNN, while all nodes in the ANNs were connected to the same (constant) number of other nodes (Fig. 2b, Additional file 1: Fig. S2b-c). This approximately scale-free nature of the KPNN derives in part from the presence of highly connected “hubs” in biological networks [45], which link many nodes with lower connectivity. As a result of these connectivity patterns, the KPNN showed higher modularity than the ANNs, which is evident from its higher sensitivity to fragmentation upon edge removal compared to fully connected ANNs, and even to sparse ANNs (Fig. 2c, Additional file 1: Fig. S2d).

The sparseness and modularity of KPNNs restrict individual hidden nodes in their access to input data via direct or indirect network connections to the input nodes. To quantify the amount of information available to each hidden node, we calculated the network reachability as the number of input nodes that a given hidden node can access through the network. Strikingly, most hidden nodes in the KPNN were connected only to a small fraction of input nodes. Such restrictions in the access to input data were much less pronounced in edge-matched sparse ANNs and absent from fully connected ANNs (Fig. 2d, Additional file 1: Fig. S2e).

In summary, the network structure of KPNNs deviates from that of generic ANNs by incorporating key properties of biological networks, such as a sparse modular architecture and hierarchy-skipping shortcuts. KPNNs have fewer free parameters that are optimized by deep learning. Moreover, every node and every edge within a KPNN has a corresponding biological interpretation. The characteristic network architecture of KPNNs is therefore expected to benefit their biological interpretability.

### An optimized learning method enhances KPNN interpretability

Complementing the characteristic network structure of KPNNs, we developed an optimized learning method that enhances the interpretability of trained KPNN models. The learning method for KPNNs resembles those commonly used for deep learning on ANNs. In a nutshell, edge weights are iteratively updated until they reflect the relationship of input data (i.e., single-cell RNA-seq profiles) to the network output (i.e., class values representing cell states or environmental exposures) throughout the structure of hidden nodes. Subsequently, we calculate hidden node weights from the edge weights, and we interpret these node weights as measures of importance of the corresponding signaling protein or transcription factor in the investigated biological system.

When we applied generic deep learning (as described in detail in the “Methods” section) to KPNNs instead of ANNs, we obtained high prediction accuracies (Additional file 1: Fig. S1b-c). However, we identified three recurrent challenges to interpreting the learned models: (i) low reproducibility in the presence of network redundancy, (ii) lack of quantitative interpretability (i.e., node weights fail to quantitatively reflect the predictive power of individual nodes), and (iii) uneven connectivity inherent to biological networks, which affects node weights independent of training data. To address these issues, we conceptualized and validated several modifications of the learning method, which jointly enhance the interpretability of KPNNs.

**Fig. 3:**
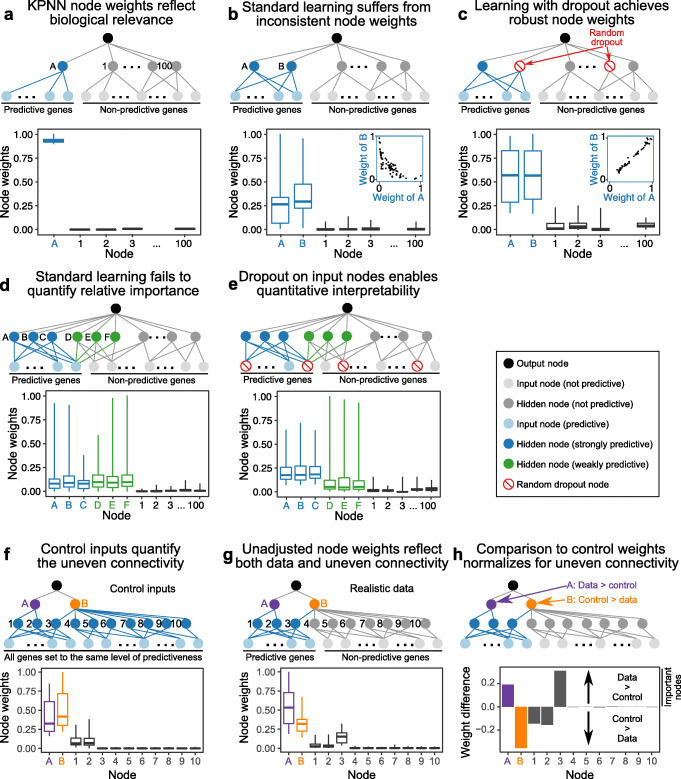
Optimized learning methodology for KPNNs. **a** Node weights reflect predictiveness in a simple network. (Top) Simulated network with one hidden node (node A) that is connected to several predictive input nodes, each representing one gene. (Bottom) Learned node weights identify node A as predictive. **b** High variability of node weights for two redundant nodes based on generic deep learning. (Top) Network with two hidden nodes (A and B) connected to predictive input nodes. (Bottom) Node weights distinguish predictive from non-predictive nodes, but there is a negative correlation of node weights for the two redundant nodes (inset). **c** Dropout reduces variability and increases robustness of node weights. (Top) The same network as in panel b, trained with dropout on hidden nodes. Dropout nodes are randomly selected at each training iteration. (Bottom) Learning with dropout results in robust and highly correlated weights (inset) for the two redundant nodes. **d** Node weights of weakly and strongly predictive nodes using generic deep learning. (Top) Network with three strongly predictive hidden nodes (A–C, connected to multiple predictive input nodes) and three weakly predictive hidden nodes (D–F, connected to one predictive input node). (Bottom) Node weights do not separate highly predictive from weakly predictive nodes when using generic deep learning. **e** Learning with input node dropout distinguishes between highly predictive and weakly predictive hidden nodes. (Top) The same network as in panel d, trained with dropout on input nodes. (Bottom) Node weights separate highly predictive from weakly predictive nodes when training with input node dropout. **f** Control inputs quantify the uneven connectivity of biological networks. (Top) Network with two layers of hidden nodes (A and B; 1 to 10) and input nodes that are all equally predictive of the output. (Bottom) Node weights trained on control inputs reflect the uneven connectivity of the simulated network. **g** Node weights obtained by training on actual data reflect both the data and the uneven connectivity. (Top) The same network as in panel f, but with only a subset of input nodes being predictive. (Bottom) Node weights for the network trained on actual data. **h** Comparison of node weights for actual data and for control inputs enables normalization for uneven network connectivity. (Top) The same network as in panel g, with annotation of the effect of input data and network structure on the importance of nodes A and B. (Bottom) Differential node weights for actual data versus control inputs

First, biological networks are characterized by widespread redundancy, for example when one protein regulates another protein via two separate signaling pathways. Such redundancy can result in a lack of reproducibility [33], such that KPNNs with widely different edge weights may achieve similar prediction performance as the result of cost functions with multiple local minima. To illustrate this problem and to demonstrate our solution, we simulated a series of simple gene-regulatory networks with matched training data (see the “Methods” section for details). We first engineered a network without any node redundancy, performed model fitting, and then calculated node weights. In this redundancy-free network, node weights accurately reflected the information flow that we embedded into the network (Fig. 3a). However, when we introduced redundancy into the network and repeatedly trained the model, generic deep learning resulted in node weights that were highly variable, and node weights of redundant nodes were inversely correlated across network replicates (*R* = − 0.79, Fig. 3b). Repeated KPNN training thus yielded inconsistent and uninterpretable results when using generic deep learning.

To address this issue and to obtain robust, interpretable node weights in the presence of widespread network redundancy, we incorporated random dropout of hidden nodes into our learning method. When networks are trained with dropout, a given percentage of nodes is randomly selected and “dropped” (i.e., set to zero) for each sample and training step. Deep learning with dropout was originally proposed as a strategy to improve the generalizability of ANNs [48]. We found that random dropout of hidden nodes dramatically improved consistency of node weights across KPNN replicates, resulting in an almost perfect correlation of weights for redundant nodes (*R* = 0.99, Fig. 3c) and an improved overall correlation of node weights between network replicates (Additional file 1: Fig. S3). These results demonstrate that dropout of hidden nodes forces deep learning to spread weights across the network instead of depending on individual nodes, which improves robustness.

Second, we found that node weights obtained using generic deep learning did not adequately reflect quantitative differences in the predictive power of individual nodes. We illustrate this issue with a simulated network comprising three strongly predictive hidden nodes (each of these nodes was connected to several predictive input nodes) and three weakly predictive hidden nodes (each of these nodes was connected to just one predictive input node). Using generic deep learning, this difference was not reflected in the trained node weights (Fig. 3d). We thus introduced dropout of input nodes into the learning method (in addition to dropout of hidden nodes), thereby forcing the learning to spread weights across input nodes. As a result, we indeed observed much-improved and robust quantitative interpretability, with a clear difference in node weights between nodes with different predictive power (Fig. 3e). This result was robust toward mistakes in the network structure (false positives and false negatives), which we confirmed by shuffling a subset of randomly selected edges (Additional file 1: Fig. S4).

Third, uneven connectivity in biological networks [45, 54] affects node weights in trained KPNNs independently of the input data, for example resulting in structurally inflated weights for central, well-connected nodes. To illustrate this effect, we simulated a network with 12 hidden nodes organized into a top layer (nodes A and B) and a bottom layer (nodes 1 to 10). We constructed this network with uneven connectivity, such that one of the top-level nodes is connected to a higher number of nodes than the other (A, 2 nodes; B, 8 nodes). We then trained with control inputs that were equivalent across input nodes, simulated such that all input nodes predicted the output equally well. Networks trained with these control inputs were biased by the underlying network structure: The strongly connected node B received a higher node weight than the weakly connected node A, and the central nodes of the top layer (node A and B) received higher node weights than the peripheral nodes of the bottom layer (nodes 1 to 10) (Fig. 3f).

To normalize for uneven connectivity in KPNNs, we compared models that were trained on the actual simulated data—resulting in node weights that reflect both the simulated biological signal and the effect of uneven connectivity (Fig. 3g), to models trained on control inputs—resulting in node weights that solely reflect network connectivity (Fig. 3f). By comparing node weights between these two models, we can quantify the degree to which a given node is more or less important than expected based on the network structure. The resulting “differential node weights” thus provide a normalized measure of node importance in the training data that is adjusted for the influence of network structure on trained node weights. Differential node weights clearly distinguished the data-driven and the network-driven weights of node A (with two out of two downstream nodes predictive) and node B (with only one out of five downstream nodes predictive) in our simulation (Fig. 3h).

In summary, we introduced three methodological advances for interpretable deep learning on KPNNs: training with hidden node dropout enhances robustness of node weights in the presence of network redundancy, input node dropout distinguishes between strongly predictive and weakly predictive nodes, and the calculation of node weights based on control inputs allows us to normalize for the effect of uneven connectivity in biological networks. Together with the sparse structure of KPNNs and the fact that every node and every edge in the KPNNs has a corresponding biological interpretation, this learning method fosters interpretable deep learning on biological networks.

### KPNNs infer a regulatory model of T cell receptor stimulation

**Fig. 4:**
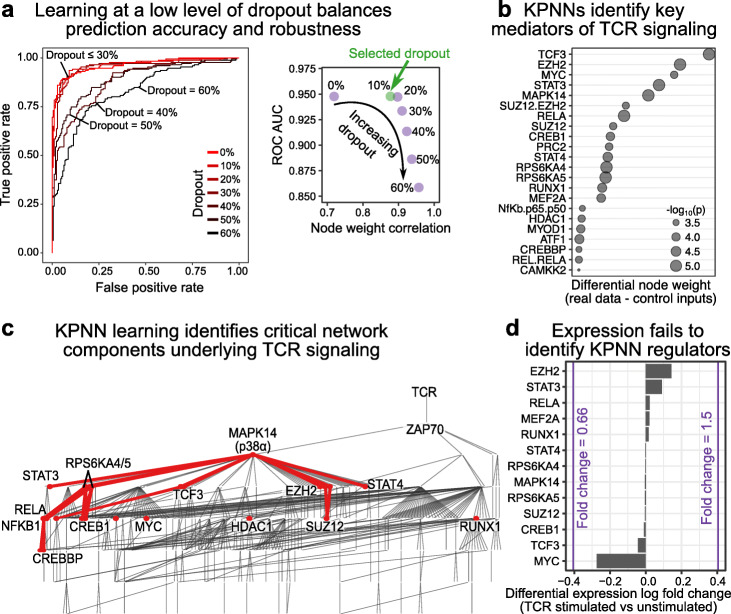
KPNN analysis of T cell receptor (TCR) stimulation. **a** Receiver operating characteristic (ROC) curves for the TCR KPNN, predicting TCR stimulation based on single-cell RNA-seq profiles with different levels of dropout. The inset shows the mean ROC area under curve (AUC) values at different dropout rates (measuring prediction performance) as well as the mean correlation across replicates (measuring network robustness). **b** Differential node weights at a dropout rate of 10%, comparing networks trained on actual data (*x*-axis) and on control inputs (*y*-axis). Nodes with *p*_adj_ below 0.05 are shown. **c** Trained TCR KPNN with the subnetwork of significantly differential nodes (*p*_adj_ < 0.05, dropout rate = 10%) highlighted in red. **d** Log fold change (LogFC) of gene expression for TCR regulators identified by the KPNN. Commonly used thresholds for differential expression (fold change = 1.5 and fold change = 0.66) are indicated in purple

When we trained the TCR KPNN with our optimized learning method, based on single-cell RNA-seq data for TCR stimulated vs unstimulated Jurkat cells [49], we obtained high prediction accuracies (Fig. 4a), comparable to those obtained by generic deep learning on KPNNs and on ANNs (Additional file 1: Fig. S1b). Moreover, we indeed observed the anticipated boost in biological interpretability of the trained KPNN model (Fig. 4b-d).

The introduction of dropout in our optimized learning method improved the reproducibility across network replicates, giving rise to highly consistent model interpretations (Additional file 1: Fig. S5). Moreover, we observed broader spreading of high node weights across the network, as illustrated by an animation of the learning process (Additional file 2: Supplementary Video 1). This gain in robustness was most pronounced between 0 and 10% dropout, with little impact on prediction performance up to 30% dropout (Fig. 4a, Additional file 1: Fig. S6). We thus selected a conservative dropout rate of 10% for further analysis, which resulted in a median node weight correlation of 0.912 (interquartile range 0.814 to 0.961), compared to 0.737 without dropout (interquartile range 0.608 to 0.854). Prediction performance was maintained with a median ROC AUC value of 0.982 with dropout (interquartile range 0.977 to 0.987), compared to 0.984 without dropout (interquartile range 0.979 to 0.987).

Node weights were normalized for uneven connectivity in the TCR signaling network by calculating differential node weights against the TCR KPNN trained on control inputs (Fig. 4b, Additional file 3: Table S1). The resulting differential node weights identified key regulators of TCR signaling such as the NF-κB1:RELA (p65:p50) transcription factor complex, its subunits RELA and NF-κB1, and the p38α MAP kinase MAPK14. Moreover, regulators with high differential node weights were enriched for MAP kinase pathways (Additional file 1: Fig. S7, Additional file 4: Table S2), which are known to be involved in TCR signaling and immune response [55].

The regulators of TCR signaling identified by our analysis formed a subnetwork within the KPNN (Fig. 4c). This subnetwork includes many regulators linked to TCR signaling, such as the transcription factors CREBBP, CREB1, and ATF [56, 57], as well as the kinases RPS6KA4 and RPS6KA5, which regulate transcription factors downstream of MAPK signaling. It further comprises the well-established T cell regulators STAT3 [58], STAT4 [59], HDAC1 [60], TCF3 [61], and RUNX1 [62, 63]. The KPNN-derived subnetwork also includes MYC [64] and the PRC2 subunits EZH2 and SUZ12 [65, 66], which are general regulators of hematopoiesis and T cell development. In contrast, ZAP70 and TCR were not part of this subnetwork—given their location at the apex of the KPNN, these two nodes received the highest possible weights for both the actual data and the control inputs.

Importantly, our analysis of the TCR dataset reinforced and validated key design decisions of our modified learning method. First, dropout dramatically enhanced the consistency of the learned node weights (Fig. 4a). Our chosen dropout rate of 10% was further validated by the fact that it resulted in the strongest enrichment for genes and proteins with a known role in TCR signaling (Additional file 1: Fig. S7). Second, normalization for the effect of uneven network connectivity was essential to obtain meaningful node weights, given that the structure of the TCR signaling network would otherwise dominate over the relevant biological information contained in the single-cell sequencing data (Additional file 1: Fig. S8).

To evaluate the effect of the introduced prior knowledge on the trained models, we compared the original TCR KPNN to a series of control KPNNs with randomized connectivity but retained global network structure, which we generated by random edge shuffling. The control KPNNs generally showed reduced prediction performance compared to the TCR KPNN (Additional file 1: Fig. S9a). The node weights in the control networks were equally dissimilar from each other as they were from the TCR KPNN, although a weak positive correlation remained (Additional file 1: Fig. S9b-c), indicating that random edge shuffling alone is insufficient to purge all biological network structure from the control networks. Indeed, when we trained the control networks on the TCR dataset or on the control inputs without normalization, they showed similar enrichment for known TCR signaling regulators as the TCR KPNN (Additional file 1: Fig. S9d-e). However, differential node weights placed the TCR KPNN among the networks with the strongest enrichment for known TCR signaling regulators (Additional file 1: Fig. S9f), and those control networks with similar enrichment were notably similar to the TCR KPNN (Additional file 1: Fig. S9g), suggesting that they retain the enrichment because of this similarity. Overall, these results reinforce the relevance of biological network structure for interpretable deep learning using KPNNs.

Finally, we benchmarked the KPNN against other machine learning algorithms, both in terms of prediction performance and biological interpretability. When we trained elastic nets, random forests, support vector machines, and neural networks on the TCR dataset, we observed high prediction performance for all methods (Additional file 1: Fig. S10a). In contrast, we found little overlap between the network-based interpretations of the KPNN and the interpretations based on input features weights that were obtained from the other machine learning algorithms (Additional file 1: Fig. S10b-c). For example, the NF-κB and MAP kinase regulators identified by the KPNN were not detected by any of the other algorithms. This observation is expected and reassuring given that KPNN interpretability seeks to uncover signaling proteins and transcription factors with cell-state-specific biological activity at the protein level; such factors are not necessarily differentially expressed at the RNA level (Fig. 4d) and therefore not detected by conventional algorithms (Additional file 1: Fig. S10d).

In summary, interpretable deep learning with KPNNs uncovered a subnetwork of TCR-related regulators relevant to the specific biological system that we investigated here. We thus exploited single-cell RNA-seq data to contextualize a much broader network of potential regulators that may be linked to TCR signaling based on public databases. We also compared KPNNs with alternative machine learning methods and found that the latter could not identify signaling proteins and transcription factors that have important regulatory roles but are not differentially expressed.

### KPNNs are broadly useful for interpretable deep learning on single-cell RNA-seq data

Having established and validated KPNNs on simulated data and on the TCR dataset, we sought to apply our method to a wider spectrum of biological questions. To that end, we obtained recently published single-cell RNA-seq datasets of cancer and immune cells from the Human Cell Atlas and other sources, and we derived a generalized KPNN that does not require prior knowledge of the receptors and signaling pathways relevant to the biological system of interest (this is in contrast to the TCR KPNN, where T cell receptor activity was designated a priori as the output node). The resulting “GEN KPNN” integrates signaling pathways and gene-regulatory interactions from public databases into a single network that is directly useful for interpretable deep learning and compatible with a broad range of single-cell RNA-seq datasets.

We constructed the GEN KPNN (Additional file 1: Fig. S11, see the “Methods” section for details) by connecting multiple cell surface receptors via shortest paths through a network of protein signaling and gene-regulatory interactions (hidden nodes) to the gene expression profiles that are measured by single-cell RNA-seq (input nodes). We introduced dataset-specific output nodes that represent the sample annotations of interest for the corresponding dataset (e.g., cell type or disease state), and we connected each of these output nodes to all cell surface receptors, reflecting the concept that phenotypic cell states can be captured by the ability of individual cells to interact with their cellular environment. The resulting GEN KPNN showed very similar network properties as the TCR KPNN (Additional file 1: Fig. S12), suggesting that it enables interpretable deep learning in the same way as described for the TCR KPNN.

First, we applied the GEN KPNN to a large reference dataset from the Human Cell Atlas (HCA) comprising 483,084 immune cells [50]. This dataset contains T cells, B cells, and monocytes, each derived from two sample sources, bone marrow and cord blood. Given that the differences between immune cell types have already been studied extensively, we focused instead on differences between cells obtained from bone marrow vs cord blood. To that end, we trained the GEN KPNN to predict cell type from gene expression, separately for single-cell transcriptome profiles of 262,895 immune cells from bone marrow and 220,189 immune cells from cord blood. The KPNN achieved high prediction performance (Additional file 1: Fig. S13a), similar to other machine learning methods previously used to predict cell types from single-cell RNA-seq data [67–70], and it enabled us to systematically compare gene-regulatory mechanisms for bone marrow and cord blood.

**Fig. 5:**
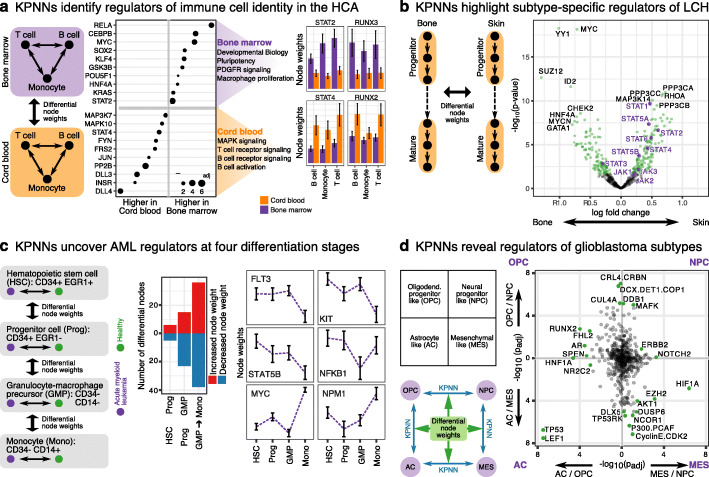
KPNN analysis of cancer and immune cells. **a** Analysis of a large Human Cell Atlas (HCA) dataset comprising B cells, T cells, and monocytes obtained from bone marrow and cord blood. (Left) KPNNs were trained as multi-class predictors separately for immune cells from bone marrow and cord blood; the fitted models were compared by calculating differential node weights. (Middle) Top 10 hidden nodes with the most differential weights between bone marrow and cord blood. Gene set enrichments were calculated against the full list of hidden nodes that carried differential weights. (Right) Differential node weights of selected proteins are shown for illustration. Error bars indicate standard error of the mean. **b** Analysis of Langerhans cell histiocytosis (LCH). (Left) KPNNs were trained separately on single-cell RNA-seq data for LCH biopsies from bone (single-system LCH) and skin (multi-system LCH), distinguishing between progenitor-like and mature LCH cells. (Right) Volcano plot comparing differential node weights between KPNNs for bone vs skin. Significant nodes are highlighted in green, JAK-STAT proteins in purple. **c** Analysis of acute myeloid leukemia (AML). (Left) KPNNs were trained to distinguish between leukemic and normal cells at four stages of hematopoietic development based on single-cell RNA-seq data, and node weights of KPNNs trained on consecutive states were compared. (Middle) Number of differential nodes comparing consecutive states. (Right) Weights of selected nodes over the four stages of hematopoietic development. Error bars indicate standard error of the mean. **d** Analysis of glioblastoma. (Left) Four glioblastoma subtypes were arranged into quadrants as in the original publication. KPNNs were trained to distinguish pairs of glioblastoma subtypes based on single-cell RNA-seq data, and differential node weights were calculated for each comparison of trained KPNNs. (Right) Scatterplot showing differential node weights between glioblastoma subtypes. For better visualization, the axes were capped at a −log_10_(*p*_adj_) value of 7.5, which affected TP53 and LEF1 (shown at the bottom left). Specific nodes of interest are highlighted in green

Hidden nodes with higher weight for bone marrow-derived cells comprised key regulators of cell fate, including SOX2, KLF5, KLF4, MYC, KRAS, and POU5F1, and they were enriched for key aspects of development, proliferation, and pluripotency (Fig. 5a, Additional file 5: Table S3, Additional file 6: Table S4). In contrast, hidden nodes with higher weight for cord blood-derived cells comprised regulators associated with functions of mature immune cells, including multiple MAP kinases and protein phosphatases, and they were enriched for pathways such as T cell receptor signaling, B cell receptor signaling, B cell activation, and innate immune response. Moreover, we observed a striking difference between STAT2 (more important in bone marrow) and STAT4 (more important in cord blood). Both proteins are part of the JAK-STAT signaling pathway [71, 72], and STAT2 is well-known for its role in type I interferon signaling [72], while STAT4 is primarily linked to IL-12 signaling and T helper cells [73]. The KPNN thus indicates that STAT2 plays a more prominent role in developing cells of the bone marrow, while STAT4 is more relevant for mature cord blood-derived cells.

We observed similar differences between RUNX3 (more important in bone marrow) and RUNX2 (more important in cord blood), consistent with reports that RUNX3 is critical for early T cell development [74, 75], while RUNX2 has been implicated in more mature roles of T cells including thymic development and viral response [76, 77]. Our KPNN analysis thus identified more developmentally immature B cells, T cells, and monocytes in bone marrow compared to cord blood. Importantly, these observations were not due to differences among hematopoietic stem cells, which were not included in our analysis. Rather, they capture gene-regulatory differences among the bulk of B cells, T cells, and monocytes between these two immune cell compartments.

Second, we explored the utility of the GEN KPNN for analyzing disease-associated cell states in Langerhans cell histiocytosis (LCH), a rare developmental disorder that combines relevant aspects of a cancer and of an autoinflammatory disease [78]. Based on a recently published single-cell RNA-seq analysis of LCH [51], we trained the GEN KPNN to distinguish between progenitor-like and mature LCH cell populations described previously. We obtained separate KPNN models for (i) bone-derived LCH cells from patients with relatively benign single-system disease and (ii) skin-derived LCH cells from patients with aggressive multi-system disease. The KPNN achieved high prediction performance for this dataset (Additional file 1: Fig. S13b), thereby enabling a meaningful comparison of hidden node weights between the two fitted models (Fig. 5b and Additional file 7: Table S5). Several of the identified regulators of LCH cells in bone were related to cell growth and differentiation, including YY1, SUZ12, MYC and MYCN, and GATA1. In contrast, LCH cells in the skin were characterized by high regulatory importance assigned to signaling proteins, including almost all JAK and STAT proteins (Fig. 5b). These results identify inflammatory signaling through the JAK/STAT pathway as a potential contributor to aggressive multi-system LCH in the skin.

Third, we investigated cellular differentiation hierarchies in a single-cell RNA-seq dataset of acute myeloid leukemia (AML), a hematopoietic cancer that is characterized by extensive cellular heterogeneity [52]. We trained the GEN KPNN to distinguish leukemic cells from healthy controls at four stages of myeloid differentiation: Hematopoietic stem cells (CD34+EGR1+), progenitor cells (CD34+EGR1−), granulocyte-macrophage precursors (CD34−CD14−), and monocytes (CD34−CD14+). The KPNN achieved high prediction performance for distinguishing between leukemic and non-leukemic cells at all four stages (Additional file 1: Fig. S13c). Comparing the hidden node weights of the trained KPNNs, we identified an iterative increase in the number of differentially regulated factors for each stage of the differentiation hierarchy (Fig. 5c and Additional file 8: Table S6). Moreover, monocytes showed very different regulatory patterns compared to the three types of progenitor cells. For example, we found that the receptor tyrosine kinase FLT3 had a greater role in leukemic progenitors (but not in leukemic monocytes) compared to their normal counterparts, consistent with the known oncogenic role of FLT3 and its association with poor disease prognosis [79]. Similar patterns were observed for the proto-oncogenes KIT and STAT5B, which are closely linked to AML-specific signaling via FLT3 and the JAK/STAT pathway [80, 81]. In contrast, leukemic monocytes were characterized by increased importance of the transcription factors MYC, NFKB1, and NPM1, all of which have been implicated in AML biology [82–84]. These results indicate striking regulatory differences between progenitor cells and differentiated cells in AML.

Fourth, we focused on glioblastoma, a brain cancer characterized by molecularly defined subtypes that tend to co-exist in the same tumors. Based on recently published single-cell RNA-seq data of glioblastoma patient samples [53], we trained the GEN KPNN to differentiate between four cell states: astrocyte-like cells (AC), mesenchymal-like cells (MES), neural progenitor-like cells (NPC), and oligodendrocyte progenitor-like cells (OPC). We observed high prediction accuracy for all pairwise comparisons (Additional file 1: Fig. S13d). The trained KPNN models recapitulated characteristic molecular differences between glioblastoma cell states (Fig. 5d, Additional file 9: Table S7, Additional file 1: Fig. S13d), including an association of HIF1A regulatory importance with MES cells [85] and of ERBB2 with NPC cells [86]. We also uncovered novel associations such as an elevated regulatory importance of NOTCH signaling [87] in MES and NPC, and of RUNX2 [88–90] in AC and OPC. Furthermore, OPC and NPC were characterized by high regulatory importance of protein degradation through CUL4A and its complexes, AC and MES showed a strong role of DLX5 [91] related signaling, and AC was characterized by pronounced regulatory importance of TP53 [92] and LEF1 [93, 94]. More generally, the trained KPNNs constitute regulatory network models for all four glioblastoma cell subtypes, which confirmed and extended previous claims of greater pairwise similarity between AC and MES, and between OPC and NPC, than for the other combinations of cell states.

In summary, we created a generalized KPNN that incorporates cell surface receptors, signaling pathways, and gene-regulatory interactions into a single network that is directly applicable to a broad range of biological questions, and we demonstrated its utility on four single-cell RNA-seq datasets. We found that interpretable deep learning with KPNNs was practical even for large single-cell atlases, achieved high prediction accuracies for distinguishing between cell states, and uncovered characteristic regulatory differences for a range of applications in immunity and cancer biology. These results establish KPNNs as a widely useful method for biological discovery, as it combines the interpretability of biological networks with the power of deep learning.

## Discussion

Deep learning has great potential for predictive analysis of biological phenomena, but its utility for scientific discovery is reduced by the “black box” character of the learned models. Indeed, even for highly predictive deep learning models, it is often difficult to derive human-understandable descriptions of the patterns that enabled successful predictions. To address this shortcoming, interpretable deep learning has emerged as an active area of machine learning research [95, 96], which has focused primarily on the ex post interpretation and reverse-engineering of trained models. For example, it is possible to visualize what ANNs trained for image recognition “see” when they classify a picture as a cat or a house [97–99], or to extract DNA sequence motifs that are predictive of tissue-specific enhancer regions [31, 100]. In the current study, we propose, implement, and validate a very different approach. We show that deep learning can be performed on biological networks, where each node corresponds to a protein or a gene, and each edge corresponds to a regulatory interaction. Our study thus demonstrates deep learning with ex ante, built-in, molecular interpretability.

Several technologies converged to enable interpretable deep learning on biological networks with single-cell RNA-seq data as input. First, two decades of research into systems biology and regulatory networks have established large databases of signaling pathways and gene-regulatory interactions, built on high-throughput experiments and on manual curation of many individual mechanistic studies. Second, recent progress in single-cell sequencing makes it possible to obtain transcriptome profiles for many thousands of single cells, thus providing ample experimental data for “contextualizing” these biological networks—at a sufficiently large scale for deep learning to play out its strengths. Third, deep learning has emerged as a method for inferring hidden states in deep neural networks based on large training datasets, potentially allowing us to infer unobserved states in complex biological networks. By combining compendia of potential regulatory interactions from public databases with single-cell RNA-seq data for the investigated biological system, deep neural networks may indeed be able to unravel those regulatory mechanisms that are relevant to the biological question of interest.

Our proof-of-concept for interpretable deep learning on biological networks comprises two key components: the knowledge-primed neural networks (KPNNs), which enable interpretability by providing a representation of biological networks that can be fitted by deep learning; and the optimized learning method, which provides a workflow for training the KPNNs based on single-cell RNA-seq data in a way that enhances their interpretability.

We derived and validated an application-specific TCR KPNN for T cell receptor signaling and a generalized GEN KPNN for a broad range of applications. These KPNNs model the signaling pathways of a cell by connecting cell surface receptors via signaling pathways and transcription factors (hidden nodes) to their target genes (input nodes corresponding to gene expression profiles obtained by single-cell RNA-seq). We demonstrated the feasibility of deep learning on these KPNNs and found that the prediction performance was comparable to that of ANNs and other machine learning methods. A comparison of network structures showed that KPNNs are structurally distinct from ANNs (greater sparseness, higher modularity), which likely enhances their interpretability by restricting the KPNN training to biologically meaningful models. Indeed, we found that trained KPNNs showed more relevant enrichments than structurally similar networks with randomly shuffled edges.

We also optimized the learning method to address three obstacles to interpretable deep learning on biological networks: (i) *Hidden node dropout*: Short-term removal of random hidden nodes during training conferred robustness to the KPNN interpretations in the context of redundant signaling pathways; (ii) *Input dropout:* Short-term removal of random parts of the input data enhanced the quantitative interpretability of node weights; and (iii) *Correction for network structure*: KPNN training on simulated control inputs allowed us to adjust for uneven connectivity inherent to biological networks. These three methodological optimizations enhanced the biological interpretability of KPNNs both on simulated and on real data. We anticipate that similar optimizations will also be relevant to other studies in the growing field of interpretable deep learning [36, 39, 40, 42, 101].

As a proof-of-concept for interpretable deep learning on biological networks, we applied our method to five large single-cell sequencing datasets covering a range of biological applications. First, we investigated TCR signaling in vitro, and the trained KPNNs indeed recapitulated key aspects of what is known about TCR signaling, contextualized to the concrete biological model (Jurkat cells stimulated by CD3/CD28 antibodies). Second, we demonstrated the scalability of our method on a large immune dataset from the Human Cell Atlas (483,084 single-cell transcriptomes), where we discovered unexpected regulatory differences between mature immune cells obtained from bone marrow vs cord blood. Third, we analyzed single-cell RNA-seq profiles from patients with Langerhans cell histiocytosis, and we identified JAK/STAT signaling as a potential contributor to an aggressive disease course. Fourth, we compared leukemic and normal cells at multiple stages of hematopoietic differentiation, uncovering stage-specific regulators in acute myeloid leukemia. Fifth, we inferred subtype-specific gene-regulatory networks in tumor samples from patients with glioblastoma. In all of these cases, the KPNNs discovered both known and novel regulators based on the single-cell RNA-seq data.

These applications—together with our extensive analyses of simulated data—support the validity and practical utility of interpretable deep learning using KPNNs. Nevertheless, there are potential limitations that should be considered by potential users of our method. First, KPNNs currently require directed acyclic graphs, in order to be compatible with deep learning algorithms. We have developed a procedure that derives high-confidence acyclic KPNNs from biological networks with cycles, but explicit modeling of cycles within KPNNs could be advantageous. Second, our current KPNNs are restricted to discovering and contextualizing regulatory mechanisms that have previously been annotated in public databases, while entirely novel and unexpected interactions may be missed. Third, KPNNs may suffer from false positives and false negatives in the annotation data, although we found that the results were robust toward shuffling a limited number of edges in simulated networks. Fourth, the specificity of the KPNNs (i.e., ability to confidently exclude regulatory mechanisms that are not relevant in the investigated biological system) depends on the quality of the single-cell RNA-seq training data, and noisy or biased experimental data may affect the performance of our method. Fortunately, KPNNs appear to cope well with the inherently low coverage and frequent experimental dropouts in single-cell RNA-seq data, and we obtained comparably robust results on in vitro as well as ex vivo profiles of human cells.

Beyond these practical considerations, our study raises conceptual and methodological challenges and opportunities. Most notably, it poses the question how interpretability should be defined more formally in the context of interpretable deep learning on domain knowledge networks. Here we explored the hypothesis that the structural analogy between KPNNs and biological networks, together with our optimized training method, will result in trained KPNN models that capture key aspects of the regulatory mechanisms at work in the biological system of interest. We empirically tested this hypothesis based on simulated data and in multiple biological applications of KPNNs, and our results support the practical utility of our method. The ability to infer molecular mechanisms distinguishes KPNNs from most methods for single-cell RNA-seq data analysis [102, 103]. More generally, KPNNs provide a deep learning-based framework that complements existing bioinformatic methods for identifying key transcription factors [104–106] and other regulatory proteins [107–109] from single-cell gene expression data [110, 111]. KPNNs are also complementary to well-established paradigms for mathematical modeling and computational simulation of signaling pathways and gene regulatory networks [46, 47, 112].

It will be interesting to explore in more detail how KPNNs compare to and potentially complement other machine learning methods. Based on our observations, KPNN interpretability—with its focus on inferring the regulatory importance of signaling proteins and transcription factors—is conceptually and empirically distinct from existing methods for ex post interpretability based on feature weights for (observed) gene expression levels. Important signaling proteins and transcription factors identified by KPNNs were rarely differentially expressed at the RNA level, and many of them are therefore undetectable by alternative methods. As illustrated on simulated data with known ground truth, the node weights in the KPNNs capture regulatory importance at the protein level, suggesting that KPNNs may help derive mechanistic hypotheses about the role of signaling proteins and transcription factors from single-cell RNA-seq datasets. In the future, it will be interesting to compare regulatory mechanisms inferred by KPNNs to those established using high-throughput experimental approaches such as CRISPR screening or phosphoproteomics and to validate the most interesting regulatory mechanisms identified by KPNNs with detailed mechanistic studies.

Finally, KPNNs are loosely related to graph neural networks (GNNs). GNNs use domain knowledge to connect input nodes in networks, and input node values are iteratively updated by sharing information between neighboring nodes prior to prediction [113, 114]. In genome biology, GNNs have been used to predict clinical attributes from gene expression [115]. While GNNs and KPNNs both build on networks that capture prior domain knowledge, GNNs use network knowledge to share information between input nodes (within the input layer) in order to improve prediction performance. Interpretability of GNNs is thus restricted to the updated inputs. In contrast, KPNNs use network knowledge to connect hidden nodes (between layers) in order to achieve deep, multi-layer interpretability of the trained model. Toward this end, KPNNs exploit the analogy between biological regulation (signals are transduced from receptors via signaling proteins to transcription factors, which control gene expression) and feed-forward neural networks (output nodes, hidden nodes, input nodes), thus providing a new way of performing deep learning using domain-specific graphs/networks.

## Conclusions

This study provides a general framework and initial proof-of-concept for interpretable deep learning on biological networks. We demonstrated the utility of knowledge-primed neural networks (KPNNs) for the molecular interpretation of single-cell RNA-seq data, which is an exciting and highly active research area. Moreover, given the broad interest in networks for describing biological mechanisms, we expect that our use of deep learning on biological networks will also be relevant in other areas of biology and medicine, for example analyzing metabolome/proteome data, biochemical reaction networks, cellular differentiation, or even brain circuits.

## Methods

### Construction of a general regulatory network for human cells

Knowledge-primed neural networks (KPNNs) integrate multiple signaling pathways and gene-regulatory interactions into a single network that can be fitted by deep learning. As the biological basis of these KPNNs, we integrated large-scale, genome-wide datasets of transcription factor target genes and protein signaling interactions from several public databases into a broad network model of the potential regulatory space in human cells. This general regulatory network model is represented as a directed graph.

#### Transcription factors

Genes constitute the input nodes of the KPNNs, and their expression levels are measured by single-cell RNA-seq. Transcription factors constitute part of the KPNNs’ hidden nodes, and their regulatory importance is inferred by deep learning. To connect the genes to the transcription factors that may regulate their expression, we obtained transcription factor/target gene pairs from the Harmonizome [116] database (as of 3 July 2017), including the following data sources: ENCODE, CHEA, ESCAPE, MotifMap, and TRANSFAC (including both manually curated and predicted binding sites). Further transcription factor/target gene pairs were obtained from the TTRUST [117] database (as of 3 July 2017), which we mapped to gene names using an NCBI resource (ftp://ftp.ncbi.nlm.nih.gov/gene/DATA/gene_info.gz, as of 10 October 2016). Different datasets were combined by counting the number of datasets that support a connection for each transcription factor/target gene pair. To prioritize experimental over computational evidence, weights of 1.0 and 0.3 were used for experimental and computational support, respectively. For each gene, the transcription factors with the largest weighted number of datasets supporting the connection to the gene were retained. As a result of this procedure, 745 genes were targeted by more than 25 transcription factors; these genes were removed from the analysis to avoid biasing the network by including promiscuous or false-positive regulatory interactions.

#### Signaling pathways

Signaling proteins also constitute part of the KPNNs’ hidden nodes. To connect signaling proteins to their target proteins (transcription factors or other signaling proteins), we obtained a comprehensive dataset of protein signaling interactions, protein complexes, and protein family information from the SIGNOR [118] database (as of 3 July 2017), which is a large, manually curated database of directed signaling interactions. To focus this network on signaling pathways that connect proteins, we removed interactions of transcriptional regulation, guanine nucleotide exchange factor, transcriptional activation, post-transcriptional regulation, and transcriptional repression, and we removed all nodes that were not proteins, complexes, or protein families, as well as nodes from databases other than UniProt [119] and SIGNOR. All retained interactions were aggregated into a directed graph of potential regulatory interactions. To capture interactions between complex members and annotations of protein families, we further linked family members to nodes representing protein families, and protein complex members to nodes representing complexes. This was done using edges in both directions, thus enabling protein complex members to regulate their target complexes and vice versa.

### Construction of application-specific KPNNs for deep learning

From the general regulatory network described in the previous section, application-specific KPNNs are derived as follows: (i) define the cell surface receptor(s) expected to be most relevant for the biological phenomenon of interest, (ii) extract a directed acyclic graph that connects the selected receptor(s) to all reachable transcription factors, and (iii) connect transcription factors to their target genes. The resulting graphs are used for interpretable deep learning by reversing the cascade: Measured gene expression values (input nodes) predict the regulatory importance of connected transcription factors (hidden nodes), which predict the regulatory importance of their connected signaling proteins (hidden nodes), which then predict the regulatory importance and activation states of surface receptors, which finally predict the output (phenotype) of the specific dataset. In this study, two KPNNs were constructed: (i) a KPNN specific to TCR signaling (TCR KPNN), which seeks to predict TCR stimulation from gene expression, and (ii) a generalized KPNN (GEN KPNN) that can be used to predict cell states from single-cell RNA-seq datasets independent of specific receptors.

#### TCR KPNN

To construct the KPNN for TCR signaling, we selected the TCR node (SIGNOR-C153) as the output node of the network and calculated shortest paths to all reachable transcription factors in the general regulatory network using the function *all_shortest_paths* from the igraph package (version 1.1.2) in R (version 3.2.3). These paths were then combined, resulting in a directed acyclic graph. Finally, transcription factor/target gene pairs were used to connect each transcription factor to its target genes (input nodes).

#### GEN KPNN

To construct a generalized KPNN that does not require prior knowledge of the most relevant receptors and signaling pathways for a given application, we introduced output nodes that represent sample annotations of interest in a given dataset (e.g., cell type or disease state). Output nodes were adapted to the specific biological question and dataset: In the HCA dataset, three output nodes were used to represent B cells, T cells, and monocytes. In the other three systems, one output node was used for the binary classification of (i) progenitor vs mature cells in the LCH dataset, (ii) leukemic vs normal cells in the AML dataset, and (iii) disease subtype in the glioblastoma dataset, i.e., pairwise comparisons between astrocyte-like cells (AC), mesenchymal-like cells (MES), oligodendrocyte progenitor-like cells (OPC), and neural progenitor-like cells (NPC). These output nodes were connected to all cell surface receptors, based on the pathway annotations in the SIGNOR database obtained with SIGNOR’s REST API. We then calculated shortest paths from the output nodes via the cell surface receptor nodes to all reachable transcription factors in the generic regulatory network using the function *all_shortest_paths* from the igraph package. These paths were combined, resulting in a directed acyclic graph. Transcription factor/target gene pairs were used to connect each of the transcription factors to their target genes (input nodes). This initial graph was then extended to ensure inclusion of all relevant cell surface receptors. To that end, shortest paths from individual receptors to all transcription factors were added under the condition that they do not introduce cycles. This was ensured by the following procedure: First, shortest paths from all receptors to all transcription factors were identified in the general regulatory network and transformed to an edge list. Second, edges from this list were added to the graph if the edge’s parent depth was smaller than the child depth, or if the child was not yet included in the network. Node depth was defined as the distance of a node from the output nodes. In addition, to rule out feedback loops from transcription factors to upstream protein signaling pathways, the depth of transcription factors (and their downstream nodes) was artificially set to be greater than the depth of all non-transcription factors. Finally, the two steps were repeated iteratively until no more edges could be added. This procedure thus enabled us to extend our graph by adding relevant connections, while ensuring that no cycles were introduced into the graph.

### Single-cell transcriptome datasets used for KPNN training

While the KPNNs represent the wider regulatory space potentially relevant for the application of interest, it is the deep learning on KPNNs using single-cell transcriptome data that confers specificity and identifies those parts of the KPNNs that are indeed relevant and predictive for the investigated biological system. We developed and evaluated interpretable deep learning on KPNNs using simulated transcriptome profiles with known ground truth, we validated and benchmarked our method on a biological dataset of TCR stimulation, and we demonstrated its broad applicability and utility on four additional real-world datasets.

#### TCR dataset

The TCR dataset was downloaded from GEO (GSE137554). It was generated with the CROP-seq assay [49] and single-cell RNA-seq on the 10x Genomics platform. Only unperturbed cells (i.e., those that expressed non-targeting guide RNAs) were included in the analysis. To identify the CRISPR guide RNAs in each cell, a reference genome was created by extending the human GRCh38 genome assembly with sequences of all used guide RNAs with Cell Ranger *mkref*. Sequencing data were aligned to this reference genome with Cell Ranger *count* and merged with Cell Ranger *aggr*. Barcodes with less than 500 unique molecular identifiers (UMIs), less than 200 identified genes, or more than 15% mitochondrial reads were considered low-quality and removed from the analysis. Cells expressing only non-targeting guide RNAs were selected for further analysis. Class values for TCR stimulation were assigned based on whether a cell was part of the TCR stimulated or unstimulated sample.

#### Simulated data

Data with a defined ground truth were simulated based on the TCR dataset. We averaged expression counts for the unstimulated state for each gene to generate a baseline expression profile. Next, a positive or negative twofold change was introduced in selected genes (predictive/informative genes whose expression was linearly correlated with each cell’s class value), resulting in a second (differential) gene expression profile. Finally, single-cell RNA-seq profiles of individual cells were simulated by subsampling reads from these average profiles using *rbinom* in R as described previously [120]. Two thousand single-cell RNA-seq profiles were generated, 50% of which were derived from the baseline profile and 50% from the differential profile.

#### HCA dataset

The HCA dataset [50] was downloaded from the “Census of Immune Cells” that is part of the Human Cell Atlas (https://preview.data.humancellatlas.org/), as of 31 July 2018. Low-quality cells with less than 500 unique molecular identifiers (UMIs), less than 200 identified genes, or more than 15% mitochondrial reads were removed from the analysis, and expression levels were transformed to log (TPM + 1) values. Cell types (class values) were assigned based on the expression levels of cell-type-specific marker genes. Marker gene expression for CD79A and CD19 was used to identify B cells; CD3D, CD3G, and IL32 to identify T cells; and CD14 and CST3 to identify monocytes. Cells with log (TPM + 1) values above 4 for any of the listed marker genes were assigned to the respective cell types. Cells assigned to none of the cell types (which comprises all other cell types) or to multiple cell types (including cell duplicates) were removed from the analysis. Using this procedure, 483,084 from a total of 628,630 cells were uniquely assigned to one of the three cell types.

#### LCH dataset

The LCH dataset [51] was downloaded from GEO (GSE133704). Single-cell annotations were downloaded from http://www.medical-epigenomics.org/papers/LCH_hierarchy/data/lch_10x_meta.csv.gz as of 1 October 2019. Cells in subsets 1, 2, and 3 were labeled as progenitors, and cells in subsets 11, 13, and 14 were labeled as mature LCH cells. Bone and skin were annotated based on the biopsy source.

#### AML dataset

The AML dataset [52] and its single-cell annotations were downloaded from GEO (GSE116256). Cells were labeled as leukemic (cancer) or normal (healthy) cells based on the predictions of malignancy (refined predictions), which were extracted from the single-cell annotations downloaded from GEO. In addition, cells were labeled according to cell type based on single-cell annotations: hematopoietic stem cells (HSC: CD34+EGR1−), progenitor cells (Prog: CD34+EGR1+), granulocyte-macrophage precursors (GMP: CD34−CD14−), and monocytes (Mono: CD34−CD14+). Promonocytes (Promono) were also included in the initial analysis but were discarded given the low prediction performance observed for distinguishing between cancer and healthy cells.

#### Glioblastoma dataset

The glioblastoma dataset [53] was downloaded from GEO (GSE131928). Single-cell annotations were downloaded from the Broad Institute’s Single Cell Portal (http://singlecell.broadinstitute.org, dataset SCP393) as of 31 October 2019 and used to assign cells to the four subgroups identified in the original publication: astrocyte-like cells (AC), mesenchymal-like cells (MES), neural progenitor-like cells (NPC), and oligodendrocyte progenitor-like cells (OPC). A cutoff of absolute “Relative meta-module scores” smaller than 0.5 was used to remove cells that were not clearly assigned to one class.

### Generic deep learning methodology and implementation

Deep learning was performed on the application-specific KPNNs with the single-cell transcriptome profiles as training data, using a three-step workflow: (i) processing of input data, (ii) training of the KPNN or ANN as a deep neural network, and (iii) evaluation of prediction performance on unseen test data. This workflow was implemented in a custom Python (version 2.7.6) program, using the Python libraries tensorflow [121] (version 1.3.1), pandas (version 0.19.2), scipy (version 0.14.0), and numpy (1.13.2). The software implementation has also been tested successfully in a more recent version of Python (version 3.7.3). It requires three inputs: (i) a neural network graph (KPNN or ANN) in the form of an edge list, (ii) a file containing class values for each single cell, and (iii) a file containing transcriptome profiles for each single cell. The methods outlined below were applied for both KPNNs and ANNs, and they constitute the generic deep learning workflow that was used in this study.

#### Processing of input data

Input data were split into training set (60% of samples), validation set (20%), and test set (20%) using numpy *random.choice*. Gene expression data were converted to log (TPM + 1) values and normalized for each gene to a maximum value of one and a minimum value of zero. Normalization factors were calculated based on the training data and then applied to the validation and test data. For the HCA dataset, minibatches were obtained using numpy *random.shuffle*.

#### Network training

Training of the neural networks (KPNNs or ANNs) is configured by a method to initialize edge weights, an activation function for hidden and output nodes, a loss function, an algorithm to minimize the loss function, and criteria to terminate training. Network training was implemented in TensorFlow, with the following setup. Edge weights were randomly initialized using TensorFlow *global_variables_initializer*. A sigmoid activation function was used for all hidden and output nodes using TensorFlow *nn.sigmoid*. The sigmoid function was chosen because of its similarity to “on” and “off” states in biological systems. The loss function to minimize was chosen as a weighed cross-entropy with L2 regularization. To calculate the loss function, cross-entropy was first calculated using TensorFlow *nn.sigmoid_cross_entropy_with_logits*. Then, to improve training in the presence of class imbalance, the cross-entropy of each sample was weighted by the number of samples of each class. The weight for each class was calculated as (1/*N*_classes_)/(*N*_*x*_/*N*_samples_), where *N*_classes_ is the number of classes, *N*_*x*_ is the number of samples of class x, and *N*_samples_ is the total number of samples. Finally, L2 regularization was calculated using TensorFlow *nn.l2_loss* and added to the weighted cross-entropy. For this loss function, the ADAM algorithm was used to minimize the regularized, weighted cross-entropy using TensorFlow *train.AdamOptimizer*. To track the learning progress, training and validation error were calculated by subtracting predicted class probabilities from true labels. Training was terminated with early stopping, which was triggered by a specific number (“patience”) of epochs without considerable learning processes (“failed epochs”). Epochs were considered as “failed epochs” if either (i) training loss plateaued (indicative of arrival at a minimum) or (ii) validation set error increased (indicative of overfitting). To be able to recover the most promising model, new models were saved during training (using TensorFlow *train.Saver)* if they achieved considerable learning progress (decreased validation set error by a given percentage) compared to the previously saved model. When a model was saved, the counter of failed epochs (“patience”) was set to zero. After learning was terminated, the most recently saved model was loaded and used to calculate test set performance.

#### Performance evaluation

Using the most recently saved model, the error on the (previously unseen) test set was assessed and receiver-operator characteristic curves were derived as the final performance metric.

#### Hyperparameters

Training hyperparameters were chosen for each dataset by inspecting the learning progress using TensorFlow’s TensorBoard, which enables live tracking of training loss, validation error, validation loss, and edge weights. The two parameters used for early stopping (minimum percent improvement on the validation set error required to save a model; number of allowed failed learning epochs) were chosen to minimize the number of iterations run in the plateau of the learning curve. The learning rate (alpha) was selected to the highest value that ensured smoothness of learning curves. The L2-loss regularization parameter (lambda) was chosen at the largest value that did not result in weights shrinking to zero.

### Optimized learning method to enhance KPNN interpretability

On top of the generic deep learning methodology (as described in the previous section), three modifications were implemented to enhance biological interpretability of the trained KPNNs: (i) dropout on hidden nodes to improve robustness, (ii) dropout on input nodes to increase quantitative interpretability, and (iii) training on simulated control inputs to normalize for uneven connectivity in biological networks. KPNNs were trained repeatedly using this optimized learning methodology, prior to extracting node weights for interpretability.

#### Dropout on hidden nodes and on input nodes

Dropout constitutes a modification of the learning algorithm where a given percentage of nodes is randomly set to zero in each training iteration and for each sample, temporarily removing the contribution of the affected nodes. Dropout was originally developed as a regularization technique to reduce overfitting [48]. Applied to KPNNs, dropout encourages the learning algorithm to spread weights across all relevant nodes, which reduces variability of node weights across network replicates (leading to improved robustness) and balances weights across input nodes (leading to node weights that more quantitatively reflect node importance). Dropout was implemented in our network training program using TensorFlow *nn.dropout,* which was applied separately to hidden nodes and to input nodes*.*

#### Normalizing for the uneven connectivity of biological networks

To normalize for the biological network structure and connectivity patterns of KPNNs, we trained the KPNNs not only on the actual single-cell transcriptome data, but also on artificial control inputs where all input nodes were set to values that are equally predictive of the class values. All input nodes carry equal importance in this scenario, hence the resulting node weights reflect only the inherent network structure of the KPNN. Control inputs thus quantify the effect of uneven connectivity on node weights and allow us to normalize for it. Control inputs were generated in the same way as the simulated single-cell transcriptome datasets used to develop our methodology (as described above), with one difference: while only a subset of inputs were selected as predictive in the simulated dataset, the control inputs were simulated such that all inputs were equally predictive. To that end, raw read counts were summed up across all cells corresponding to one class value (e.g., unstimulated cells in the TCR data) to generate an average transcriptome profile. A positive or negative (randomly selected with numpy *random.choice*) twofold change was added to all input genes to generate a second average transcriptome profile. Reads were then drawn from the two average transcriptome profiles using numpy *random.binomial* [120]*.* The number of reads drawn was based on the number of reads in each cell in the original data. For the TCR data and simulated data, the same number of cells as in the original dataset was simulated with control inputs.

### Training the KPNNs on the single-cell RNA-seq datasets

Using the optimized KPNN learning method described in the previous section, we trained and evaluated KPNNs to predict class values from single-cell RNA data in three scenarios: (i) methods development and evaluation on simulated data, (ii) validation and benchmarking on a biological dataset of TCR stimulation, and (iii) application to four additional real-world datasets addressing different biological questions. The trained KPNN models were analyzed to obtain the biological interpretations. This section describes the parameters chosen to train KPNNs for each dataset.

#### TCR dataset

Raw read counts of all 1735 cells, class values (stimulated or unstimulated), and an edge list encoding the TCR KPNN structure were provided as input to the learning program, where they were normalized and processed as described above. Hyperparameters (alpha, 0.01; lambda, 0.1) were chosen based on the inspection of learning curves using TensorBoard. Learning was stopped after 20 failed epochs. During learning, new models were saved if they reduced the validation set error of the latest stored (i.e., so far best-performing) model by at least 20%. Dropout rates from 0 to 50% were evaluated in steps of 10 percentage points. In addition, dropout was adjusted for each node based on the number of parent nodes to account for the very sparse structure of the TCR KPNN, where networks failed to converge otherwise. Specifically, nodes with only one parent were never dropped, and dropout was limited to 10% for nodes with two parents and to 30% for nodes with three parents. Network training was repeated 90 times for each level of dropout.

#### Simulated dataset

Simulated raw read counts, class values (0 and 1), and the edge lists for the simulated networks were provided as input to the learning program. Hyperparameters were set as follows: alpha of 0.001 and lambda of 0.2 in networks with one predictive node; alpha of 0.05 and lambda of 0.1 in networks with two predictive nodes; alpha of 0.05 and lambda of 0.2 in networks with three weakly and three strongly predictive nodes; alpha of 0.05 and lambda of 0.2 in the edge shuffling experiments; and alpha of 0.05 and lambda of 0.2 for comparing networks with and without control inputs. The dropout rate was set to 30% in all analyses where dropout was used. Learning was stopped after 20 failed epochs, and new models were saved if they reduced the validation set error of the most recently saved model by at least 20%. Network training was repeated 90 times for each level of dropout.

#### HCA dataset

Raw reads of all 483,084 cells, class values (B cells, T cells, and monocytes), and an edge list encoding the GEN KPNN were provided as input to the learning program. KPNNs for bone marrow and cord blood were trained separately. Marker genes used for cell type assignment were removed prior to training. Hyperparameters (alpha: 0.05; lambda: 0.1) were chosen based on the inspection of learning curves using TensorBoard. Learning was stopped after 10 failed epochs. During learning, new models were saved if they reduced the validation set error of the most recently saved model by at least 30%. Dropout rates from 0 to 40% were evaluated in steps of 20 percentage points. Learning was performed in minibatches of 1000 single cells, which were sequentially provided to the training algorithm. Differences in the parameters used to train KPNNs on the HCA and TCR data were due to the large difference in sample size (greater than two orders of magnitude), which resulted in an improved learning progress per epoch but a much longer duration of each epoch for the HCA data. Network training was repeated 90 times for each level of dropout and each tissue.

#### LCH dataset

Raw reads of 6244 cells, class values (progenitor/mature), and an edge list encoding the GEN KPNN were provided as input to the learning program. KPNNs for bone and skin were trained separately. Hyperparameters were optimized by performing a grid search on the training data (learning rate alpha: 0.0001, 0.0005, 0.001, 0.005, 0.01, 0.05, 0.1, 0.5, or 1; L2 norm lambda: 0.001, 0.002, 0.005, 0.01, 0.02, 0.05, 0.1, 0.2, 0.5, or 1). Dropout of 10% was used in all analysis. Learning was stopped after 10 failed epochs. During learning, new models were saved if they reduced the validation set error of the most recently saved model by at least 30%. Network training was repeated 100 times for each of the two biopsy sources.

#### AML dataset

Raw reads of 21,445 cells, class values (cancer/healthy), and an edge list encoding the GEN KPNN were provided as input to the learning program. KPNNs were trained separately for each cell type (HSC, Prog, GMP, Promono, Mono). Hyperparameters were optimized by performing a grid search on the training data (learning rate alpha: 0.0001, 0.0005, 0.001, 0.005, 0.01, 0.05, 0.1, 0.5, or 1; L2 norm lambda: 0.001, 0.002, 0.005, 0.01, 0.02, 0.05, 0.1, 0.2, 0.5, or 1). Dropout of 10% was used in all analysis. Learning was stopped after 10 failed epochs. During learning, new models were saved if they reduced the validation set error of the most recently saved model by at least 30%. Network training was repeated 100 times for each cell type. Promonocytes achieved limited prediction accuracy and were thus not considered for the downstream interpretation of node weights.

#### Glioblastoma dataset

TPM-normalized read counts (obtained from GEO) of 20,589 cells, class values (pairs of glioblastoma subtypes), and an edge list encoding the GEN KPNN were provided as input to the learning program. KPNNs comparing each pair of glioblastoma subtypes were trained separately. Hyperparameters were optimized by performing a grid search on the training data (learning rate alpha: 0.0001, 0.0005, 0.001, 0.005, 0.01, 0.05, 0.1, 0.5, or 1; L2 norm lambda: 0.001, 0.002, 0.005, 0.01, 0.02, 0.05, 0.1, 0.2, 0.5, or 1). Dropout of 10% was used in all analyses. Learning was stopped after 10 failed epochs. During learning, new models were saved if they reduced the validation set error of the most recently saved model by at least 30%. Network training was repeated 100 times for each comparison of subtypes.

### Calculation of node weights as a measure of node importance

Trained KPNNs models were analyzed by calculating node weights as a reflection of node importance for the KPNN-based predictions. Edge weights are readily obtainable from the network model, but they only indicate the (local) relationship of a given node to its parent node and do not capture the (global) importance of each node for the full network. To obtain informative node weights, we applied small perturbations to each hidden node separately and measured changes in network output, thus quantifying the importance of each node to the output of the network. Finally, because the sign of the resulting node weights is largely arbitrary, we take the absolute value of the node weights as a measure of the importance of each node in the trained KPNNs.

#### Calculation of node weights using induced perturbations

To obtain informative node weights, we applied a procedure analogous to numerical gradient estimation [122], which is commonly used to evaluate the effect of small numerical perturbations of edge weights on the network predictions (outputs) in order to test the calculation of backpropagation gradients. Here, instead of perturbing edge weights, we applied perturbations to nodes, thus estimating the effect that small perturbations of node outputs (activations) have on the network predictions. For each node *n*_*i*_, network predictions (class probabilities) were calculated after perturbing *n*_*i*_ twice, once by adding and once by subtracting a small factor (epsilon, which was set to 0.001 for all datasets) from the output (activation) of *n*_*i*_. The mean difference of class probabilities after additive and subtractive perturbation was then further divided by 2*epsilon to derive a normalized measure of node weight. As a result, nodes with large node weights had a greater effect on network predictions than nodes with smaller node weights, thus quantifying the global importance of each hidden node to the network.

#### Rationale for calculating absolute values of the node weights

Node outputs can impact network predictions positively or negatively, depending on the sign of their associated edge weights. In the case of input nodes, the sign of node weights can be interpreted directly with respect to network predictions (for example, “high expression of gene X predicts TCR stimulation, whereas low expression predicts no stimulation”). For hidden nodes, however, this relationship is absent because hidden nodes can assign negative or positive weights to the associated input nodes with equal outcome. For example, a negative edge weight associated with a negative node output will result in a positive number as much as a positive edge weight associated with a positive node output. The sign of node weights will thus randomly differ between replicate networks (i.e., the same network trained separately on the same input data). In contrast, the absolute magnitude of node weights reflects the importance of each node for prediction, independent of its sign. For this reason, we calculated absolute node weights, which robustly quantify the contribution of each node to the predictions.

### Statistical analysis of node weights

After the completion of training for a given KPNN, node weights were calculated as described in the previous section and exported for downstream analysis. Each KPNN was trained in multiple replicates to capture variability that results from random initiation. Node weights of the resulting trained KPNN models were then extracted to perform statistical comparisons between either (i) input data and simulated control inputs (TCR KPNN) or (ii) pairs of KPNNs (GEN KPNN). We performed statistical differential analysis on node weights of replicate networks in much the same way as it is commonly done for gene expression data. To this end, node weights were imported into R (version 3.2.3), where they were analyzed and plotted using the packages data.table (version 1.11.4), limma (version 3.26.9), ggplot2 (version 2.2.1), and pheatmap (version 1.0.10). Pearson correlations of node weights were calculated using the function *cor* in R.

#### Analysis of node weights for the TCR dataset

Differential node weights were calculated by comparing node weights obtained for actual data vs node weights obtained for control inputs, and evaluated using gene set enrichment analysis. For these analyses, only highly predictive networks with test set error lower than 0.2 were retained. Networks with dropout greater than 30% were excluded due to reduced prediction performance. To avoid biases arising from sample size differences, the number of replicate networks in each group was downsampled to that of the smallest group (*n* = 42). Node weights of all networks were normalized using limma *normalizeQuantiles*. Significance of differential node weights was tested for each hidden node using the *t.test* function. *P* values were corrected for multiple testing using the function *p.adjust* with parameter “BH”. Nodes with adjusted *P* values below 0.05 were selected as significant. Gene set enrichment analysis (GSEA) for significant proteins was performed using the function *fgsea* of the fgsea package (version 1.2.1) in a different R version (4.3.0) as required by the package. GSEA was based on the following databases: *KEGG_2016*, *NCI-Nature_2016*, *WikiPathways_2016*, *Panther_2016*, *BioCarta_2016*, and *Jensen_TISSUES*, all obtained from Enrichr [123]. In addition, to quantify the enrichment of annotated TCR signaling proteins, we used Enrichr to download all genes annotated with “TCR Signaling Pathway_Homo sapiens_WP69” from *WikiPathways_2016* as well as genes annotated with “TCR signaling in naive CD8+ T cells_Homo sapiens” from *NCI-Nature_2016* (as of December 15, 2017).

#### Analysis of node weights for the simulated dataset

Highly predictive networks with test set error below 0.1 were used to obtain node weights, which were scaled to a minimum of 0 and maximum of 1 prior to plotting.

#### Analysis of node weights for the HCA dataset

Differential node weights were calculated by comparing node weights between KPNNs trained on cells derived from bone marrow vs cells derived from cord blood. Only highly predictive networks with precision greater than 0.9 for all three cell types were used. Similar to the TCR analysis, the number of replicate networks in each group was subsampled to that of the smallest group (*n* = 48) to avoid biases arising from sample size differences. Node weights were quantile normalized using limma *normalizeQuantiles*. Differential analysis was performed with a linear model using the function *lm*, with coefficients fitted for cell type and source. *P* values were corrected for multiple testing using the function *p.adjust* with parameter “BH”. Nodes with adjusted *P* value below 0.05 were selected as significant. Enrichment analyses of significant nodes were performed using Enrichr [123] with the databases *WikiPathways_2016*, *Reactome_2016*, *Jensen_TISSUES*, *KEGG_2016*, *NCI-Nature_2016*, *Panther_2016*, and *BioCarta_2016*.

#### Analysis of node weights for the LCH dataset

Differential node weights were calculated by comparing node weights between KPNNs trained on bone versus skin. Networks with test error smaller than 0.3 were used, node weights were normalized to quantiles, and differential node weights were calculated using limma *lmFit*. Nodes with adjusted *P* value below 0.05 were selected as significant.

#### Analysis of node weights for the AML dataset

Differential node weights were calculated by comparing node weights of pairs of KPNNs trained on consecutive steps of the hematopoietic tree (HSC vs Prog; Prog vs GMP; GMP vs Mono). Node weights were normalized to quantiles, and differential node weights were calculated using limma *lmFit*. Nodes with adjusted *P* value below 0.05 were selected as significant. Promonocytes were excluded from this analysis due to low prediction performance distinguishing between cancer and healthy cells.

#### Analysis of node weights for the glioblastoma dataset

Differential node weights were calculated for two comparisons: (i) comparing the trained KPNN distinguishing OPC and NPC to the trained KPNN distinguishing AC and MES and (ii) comparing the trained KPNN distinguishing AC and OPC to the trained KPNN distinguishing NPC and MES. Node weights were normalized to quantiles, and differential node weights were calculated using limma *lmFit*. Nodes with adjusted *P* value below 0.05 were selected as significant.

### Construction of generic ANNs and structural network comparison

To empirically define the characteristics of KPNNs, we compared and benchmarked them against generic artificial neural networks (ANNs). These ANNs were constructed such that they match the corresponding KPNNs in terms of the number of input nodes, hidden nodes, and output node(s). In contrast, the distribution of edges was notably different, and the number of hidden layers was handled as a free parameter ranging from 1 to the maximum depth of the corresponding KPNN, thus resulting in multiple ANNs for each KPNN.

#### Construction of ANNs

Fully connected ANNs (fANNs) were constructed by distributing the hidden nodes equally across all layers and adding edges to fully connect adjacent layers. Moreover, all nodes of the first hidden layer were connected to all input nodes, and all nodes of the last hidden layer were connected to the output node(s). Given that these fANNs have many more edges than their corresponding KPNNs, we also generated a separate set of sparse ANNs (sANNs). These sANNs were derived from the fANNs in a way that established the same number of edges and nodes as in the corresponding KPNN. This was done in three steps that guarantee end-to-end connectivity of the resulting sANNs: First, all edges were removed from a given fANN. Second, we generated a minimal network that spans all nodes. To that end, edges were added for each pair of consecutive layers such that every node in the lower layer was connected to exactly one (randomly selected) node in the upper layer (this step also ensured that each node in the upper layer is connected to at least one node in the lower layer, since higher layers were always smaller or equal in size compared to the lower layers). Third, edges were added back randomly until the network had the same number of edges as the corresponding KPNN. The third step was carried out separately for the input layer and for the hidden layers of the network. Consequently, the resulting sANN was equivalent to the KPNN in the number of edges connecting input nodes to hidden nodes, and also in the number of edges connecting hidden nodes with each other.

#### Analysis of network structure

Structural network analysis comparing KPNNs and ANNs was performed using the igraph package in R. Outdegree was measured with the igraph function *degree*. Distance to input nodes was measured with the igraph function *distances*. The resulting distance values were used to calculate reachability, counting the number of input nodes with finite distance. To compare modularity, the network was transformed into an undirected graph using the igraph function *as.undirected*, edges to be removed were identified using the igraph function *edge.betweenness.community*, and the number of clusters was determined with the igraph function *clusters*.

### Validation of the TCR KPNN against randomly shuffled control KPNNs

To assess the effect of the prior biological knowledge that is encoded in the TCR KPNN on the trained models, we generated a series of randomly shuffled control KPNNs that resemble the overall network structure of the TCR KPNN. The control KPNNs were established by iteratively swapping edges of the TCR KPNN (“network shuffling”) while maintaining the network topology including the in-degree and out-degree of each node. We then trained and interpreted the control networks in the same way as the original KPNN, and we compared the trained KPNN models to measure the effect of network shuffling on the network interpretations.

#### Construction of control KPNNs

The TCR KPNN was randomized to generate randomly shuffled control networks using the following multi-step strategy [124], which ensures that no circles are generated: First, nodes are grouped into layers by iteratively removing leaf nodes (i.e., nodes with an outdegree of zero), such that the first nodes to remove (leaf nodes of the original network) are assigned to the lowest layer. Second, a unique index is randomly assigned to each node under the constraint that nodes in lower layers have smaller indices than nodes in the higher layers. Third, pairs of edges are swapped under the following conditions: (i) parent node index is greater than child node index in both newly added edges and (ii) the newly added edges exist neither in the original network nor in the current shuffled network. Swapping is performed once for each edge.

#### Training and interpretation of shuffled KPNNs

Control networks were trained using the same parameters and cutoffs as the original TCR KPNNs, thus ensuring comparability. Networks were trained both on real data (*n* = 90) and on control inputs (*n* = 60). For interpretation, node weights of the best performing networks were selected such that the number of trained KPNNs for each control network corresponds to the number used for the analysis of the original TCR KPNN (*n* = 42). Differential node weights (comparing KPNNs trained on real data and control inputs) were calculated as for the TCR KPNN: First, quantile normalization was performed using quantiles from the TCR KPNN analysis. Second, significantly different nodes were selected based on a *P* value cutoff that corresponds to the adjusted *P* value cutoff of 0.05 in the TCR KPNN analysis. Third, the enrichment of known TCR regulator proteins among differential nodes was calculated for each control network.

### Benchmarking of the TCR KPNN against other machine learning methods

To benchmark and compare the prediction performance and biological interpretability of KPNNs to that of established machine learning methods, we trained elastic nets, random forests, support vector machines, and neural networks to predict TCR activation on the TCR dataset. We then evaluated their prediction performance using the ROC AUC metric and extracted input feature weights as potential biological interpretations.

#### Training and interpretation of neural networks

Neural networks were trained using the keras package (2.0.6) in Python. Data were split into 80% training set and 20% test set. Activation functions used were ReLU between layers and sigmoid for the final output. Cross-entropy loss was calculated based on the output layer and optimized using the AdaGrad algorithm. Hyperparameters were tuned on training data using fivefold cross-validation with the function *GridSearchCV* of the sklearn package (0.18.1), searching a grid of learning rate (values: 10^−3^, 10^−2^, 10^−1^, and 10^0^) and L2 norm (values: 0, 10^−2^, 10^−1^, to 10^0^). Optimal hyperparameters after 100 epochs were selected to train the final model. Training of the selected model was stopped (early stopping) once learning failed to improve the cross-entropy loss on validation data (20% of training data) by 0.01 for 10 epochs. Performance was evaluated on unseen test data. For interpretation, features in the test data were individually shuffled and changes in prediction performance were recorded to obtain estimates of feature importance.

#### Training and interpretation of other machine learning methods

Additional algorithms were trained using the caret package (6.0.78) in R. Data were split into 80% training set and 20% test set. The following algorithms were included: logistic regression with elastic net penalty (glmnet package, version 2.0.13); SVMs with linear, polynomial, or radial basis function kernel (kernlab package, version 0.9.25); and random forest (randomForest package, version 4.6.12). Hyperparameters were tuned on training data using tenfold cross-validation using the caret function *train*. Feature importance of trained models was evaluated using the *varImp* function.

#### Enrichment of transcription factor target genes

To assess our ability to identify TCR signaling proteins from the feature weights of these machine learning methods, we performed enrichment analysis on the 100, 200, and 500 top-ranked features of each model against the target genes of each transcription factor. This analysis was performed using the function fisher.test in R, and the *P* values were corrected for multiple testing using p.adjust with parameter “BH”.

## Supplementary information

**Additional file 1: Figures S1 to S13** with the corresponding figure legends.

**Additional file 2: Video S1.** Effect of hidden node dropout and input node dropout on the robustness of learned weights in KPNNs. The video visualizes the learning process for the TCR KPNN, trained twice without dropout (generic deep learning) (top) and twice with dropout (optimized learning method) (bottom). Edge color and thickness reflect edge weights, with thick red lines indicating high weights and thin grey lines indicating low weights. Edge weights were transformed to absolute values prior to plotting. The networks shown were selected by first correlating edge weights between all pairs of networks, and then selecting the two networks that are closest to the median of all pairwise correlations (R = 0.78 without dropout and R = 0.86 with dropout).

**Additional file 3: Table S1.** Differential node weights for the TCR KPNN trained on actual data vs control inputs.

**Additional file 4: Table S2.** Results of gene set enrichment analysis for differential nodes from the TCR KPNN.

**Additional file 5: Table S3.** Differential node weights comparing GEN KPNNs trained to distinguish immune cell types obtained from bone marrow vs cord blood, based on data from the Human Cell Atlas (HCA).

**Additional file 6: Table S4.** Results of an Enrichr analysis for differential node weights from the Human Cell Atlas (HCA) dataset.

**Additional file 7: Table S5.** Differential node weights comparing GEN KPNNs trained to distinguish progenitor-like and mature cells obtained from bone and skin for Langerhans cell histiocytosis (LCH).

**Additional file 8: Table S6.** Differential node weights comparing GEN KPNNs trained to distinguish leukemic and normal cells at different stages of hematopoietic differentiation for acute myeloid leukemia (AML).

**Additional file 9: Table S7.** Differential node weights comparing GEN KPNNs trained to distinguish different molecular subtypes of glioblastoma.

## Data Availability

All datasets are openly available from public databases. The TCR dataset [49] was downloaded from GEO (GSE137554). The HCA dataset [50] was downloaded from the “Census of Immune Cells” that is part of the Human Cell Atlas (https://preview.data.humancellatlas.org/), as of 31 July 2018. The LCH dataset [51] was downloaded from GEO (GSE133704). The AML dataset [52] was downloaded from GEO (GSE116256). The glioblastoma dataset [53] was downloaded from GEO (GSE131928). The source code to train and analyze KPNNs (in Python and R) is available under the GNU General Public License v3.0 as a GitHub repository [125] and in archived form in Zenodo [126].
