# Supplementary material for: Knowledge-primed neural networks enable biologically interpretable deep learning on single-cell sequencing data
Source: Genome Biol. 2020 Aug 3;21:190. doi: 10.1186/s13059-020-02100-5 (PMC7397672; doi:10.1186/s13059-020-02100-5)
Supplement: Supplementary file 1 — Additional file 1: Figures S1 to S13 with the corresponding figure legends. [file 13059_2020_2100_MOESM1_ESM.docx]

**
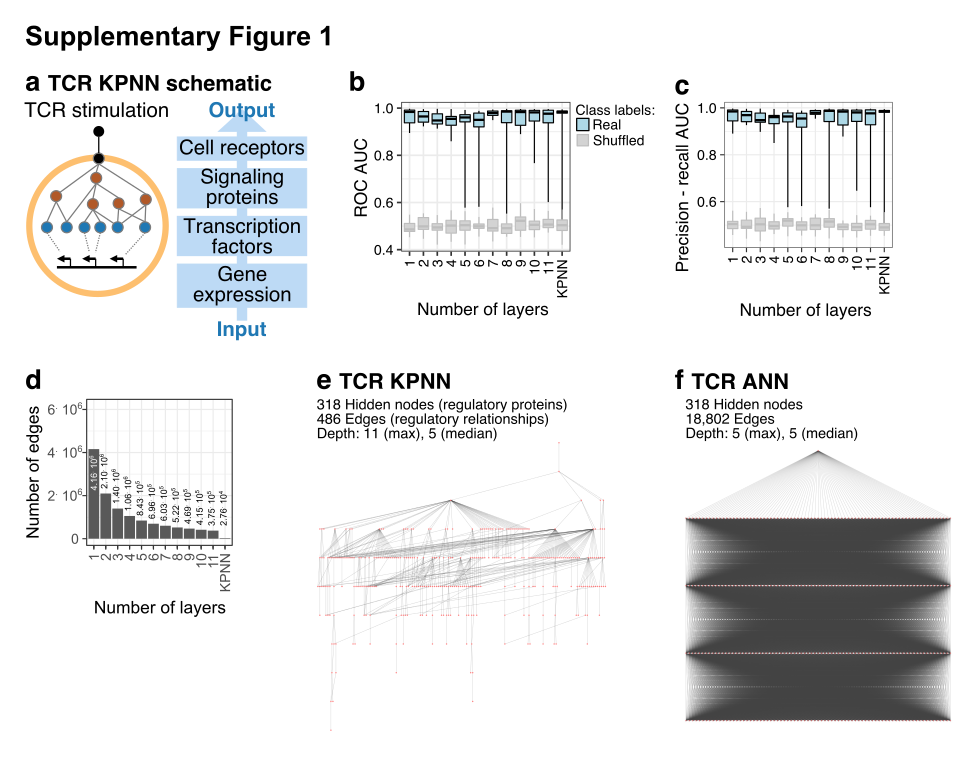
**

**Fig. S1 |** Network structure and prediction performance of the TCR KPNN and of corresponding fully connected ANNs. **(a)** Schematic outline of the TCR KPNN. Based on public databases of signaling pathways and gene-regulatory interactions, the T cell receptor (TCR) is linked via signaling pathways to transcription factors, which are further linked to their target genes. Gene expression data provide the input for the learning algorithm. The TCR is used as a single output node predicting whether or not a T cell has undergone TCR stimulation. **(b,c)** Test set prediction performance of the TCR KPNN and of the corresponding ANNs. KPNNs are compared to fully connected ANNs with the same number of nodes but different numbers of hidden layers (x-axis). Shown are receiver operator characteristic (ROC) area under curve (AUC) values (panel b) as well as the area under the precision recall curve (panel c). **(d)** Comparison of the number of edges between the TCR KPNNs and the corresponding fully connected ANNs. **(e)** Structure of the TCR KPNN, which predicts TCR stimulation from single-cell gene expression profiles. **(f)** Structure of a fully connected ANN with the same number of nodes and the same median depth as the TCR. In panel e and f, only output nodes and hidden nodes are shown, while the large number of input nodes were removed for better visualization. Network depth was calculated as the distance from the output node to input nodes.

**
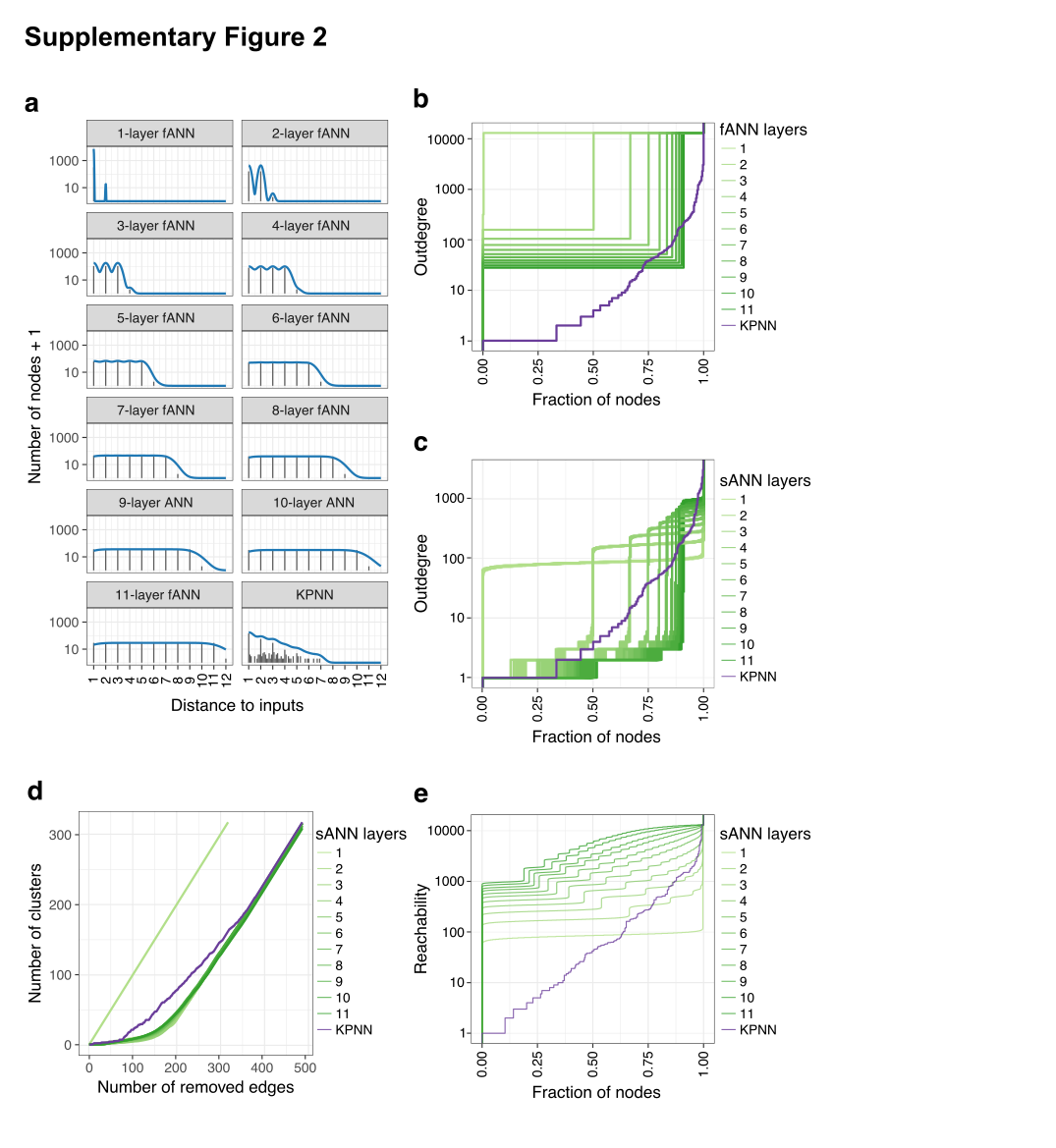
**

**Fig. S2 I** Comparison of network structure between the TCR KPNN and corresponding ANNs. The TCR KPNN is compared to fully connected ANNs (fANNs) with the same number of nodes as the KPNN, and with sparse ANNs (sANNs, n = 50 networks) in which edges were randomly removed to mimic the sparsity of the TCR KPNN. **(a)** Distribution of network distances (average distance of each hidden node to all input nodes) in the KPNN and in the fANNs. **(b)** Cumulative distribution of outdegree (number of downstream neighbors of each node) of hidden nodes in KPNN and in the fANNs. **(c)** Same as panel b, but comparing KPNNs to sANNs instead of fANNs. **(d)** Network sensitivity to targeted edge removal in the KPNN and the sANNs. Edges were removed based on their betweenness centrality values, and the number of disconnected clusters is plotted after removal of each edge. **(e)** Cumulative distribution of reachability of hidden nodes in the KPNN and in the sANNs. Reachability values measure the number of input nodes that a hidden node can access directly or indirectly (i.e., the number of inputs that are available to the hidden node).

**
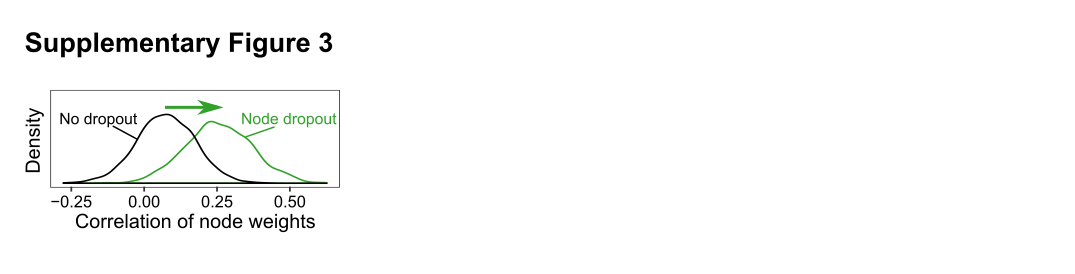
**

**Fig. S3 |** Effect of dropout on node weight correlations. Density plots are shown for the correlation of node weights learned with and without hidden node dropout.

## ****

**Fig. S4 |** Robustness of quantitative KPNN interpretability toward shuffling of network edges. (**a**) A simulated network is constructed to contain four hidden nodes with different numbers of predictive and non-predictive input nodes (genes / features) connected to each one of them. At each shuffling iteration, one edge from each node is swapped with one edge from another node (four edges from four nodes are changed in total, two are highlighted in red for illustration). (**b**) Median node weights of the four nodes over shuffling iterations.


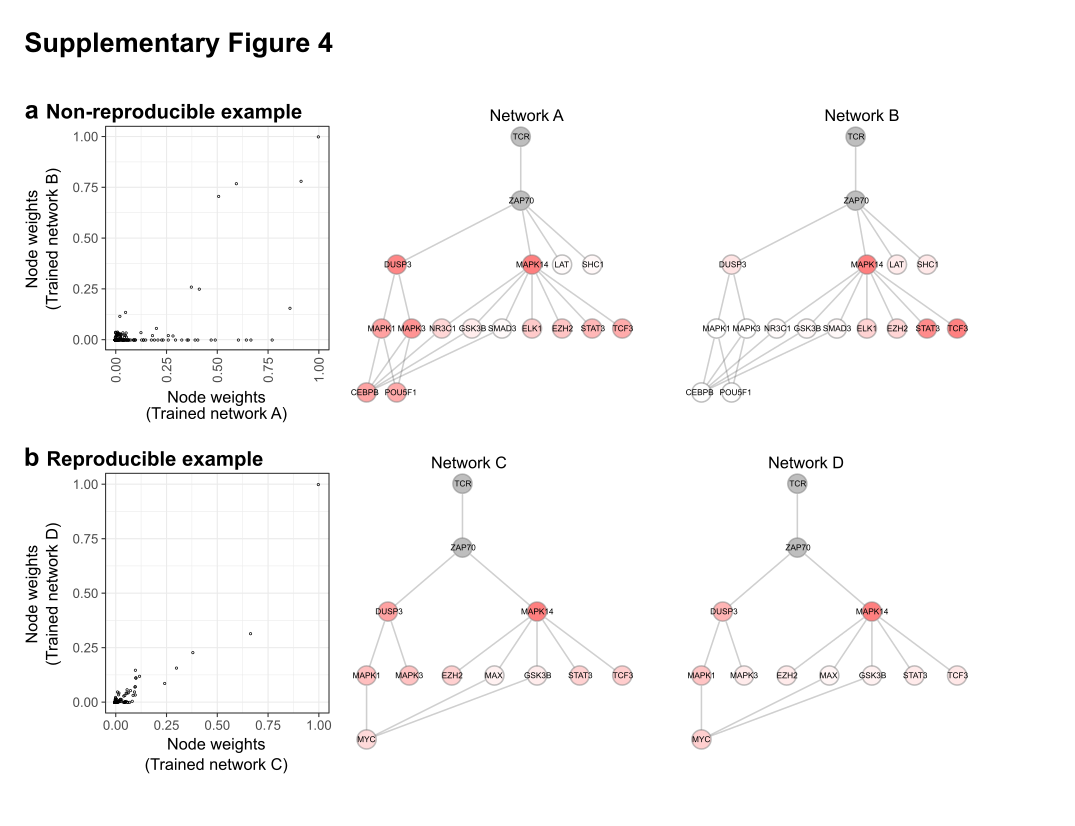


**Fig. S5 |** Detrimental effect of low reproducibility on interpretability. **(a)** Example of low correlation between two network replicates learned without dropout. The two networks produce highly inconsistent results. This is exemplified by the node weights for DUSP3, which receives high importance in only one of the two replicate networks. **(b)** Example of high correlation between two replicates learned with dropout, which resolves the inconsistencies for DUSP3 and other nodes illustrated in (a).

**Fig. S6 |** Prediction accuracy (as measured by mean ROC AUC values) of KPNNs trained with different levels of input node dropout (green) or hidden node dropout (purple). Multiple KPNNs were trained at each dropout level (n = 100), and the mean ROC AUC value of all converged KPNNs (test error < 0.2) is shown.

**
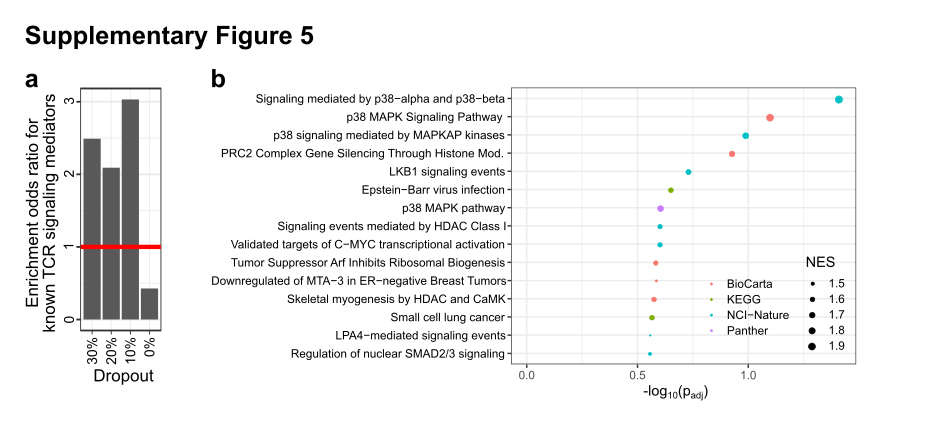
**

**Fig. S7 |** Interpretation of node weights in the TCR KPNN. (**a**) Enrichment of annotated TCR signaling mediators among significantly differential nodes at different dropout rates. (**b**) Gene set enrichment analysis of differential nodes at a dropout rate of 10%. The 15 most enriched gene sets are shown.

**
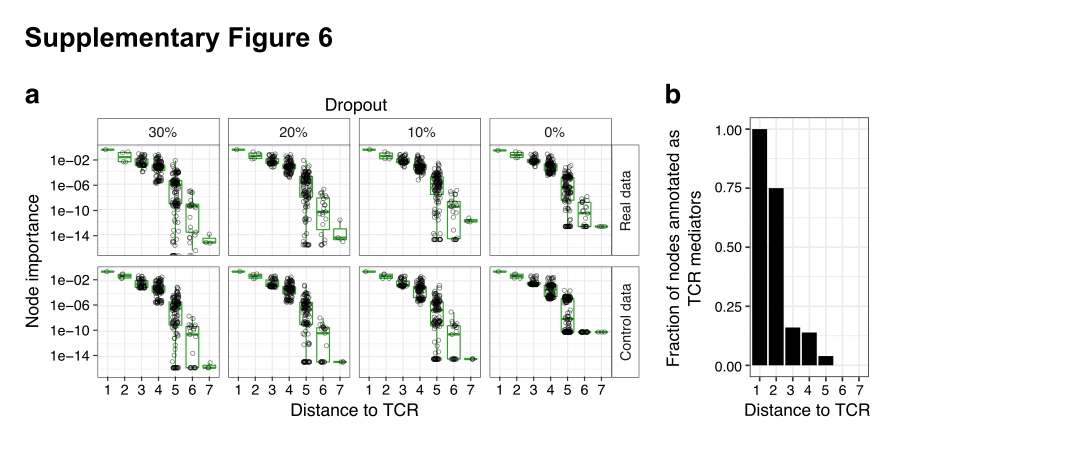
**

**Fig. S8 |** Effect of the uneven connectivity of biological networks on node weights in the TCR KPNN. (**a**) Relationship between node weights and the distance to the output node for the TCR KPNN. Hidden nodes closer to the output node have a greater effect on network output and therefore receive larger node weights not only on the actual data, but also on control inputs. (**b**) Nodes closest to the TCR in the KPNN are most strongly enriched for annotated TCR mediators.

**
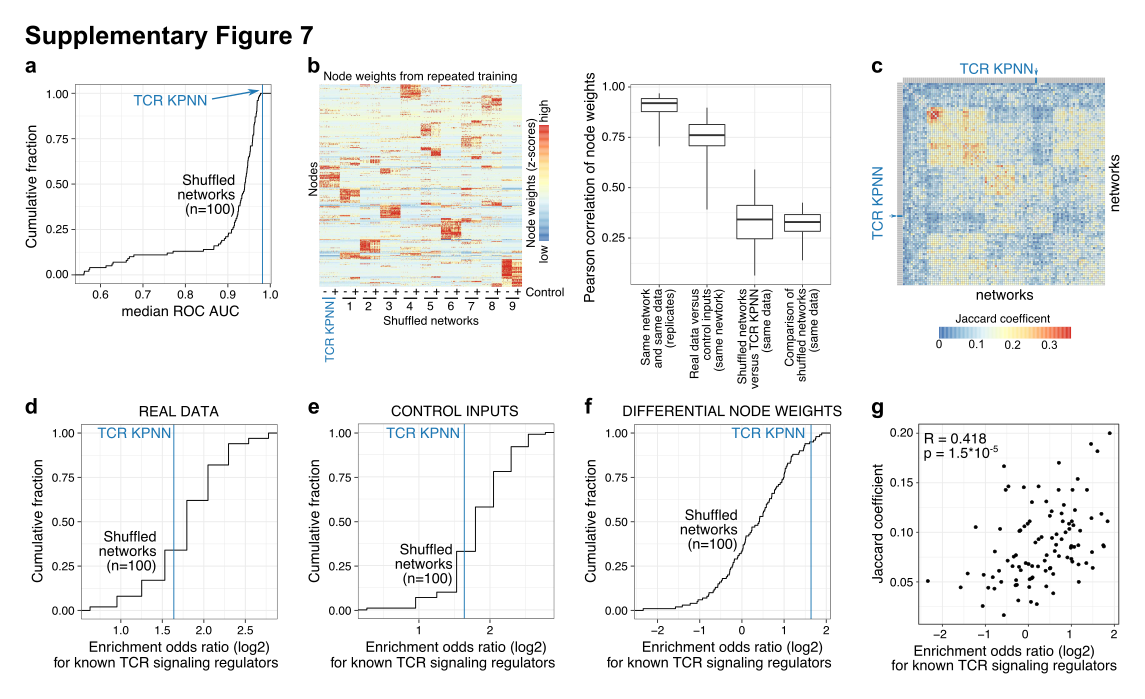
**

**Fig. S9 |** Validation of KPNN interpretability by network shuffling. The trained TCR KPNN is compared to randomly edge-shuffled control networks (n = 100). **(a)** Prediction performance of the TCR KPNN and randomly edge-shuffled control networks. ROC AUC values for the control networks are shown as a cumulative distribution, with the ROC AUC value for the TCR KPNN indicated by a blue line. **(b)** Comparison of node weights between the TCR KPNN and control networks. (Left) Heatmap of node weights for the TCR KPNN and nine randomly selected control networks. Node weights shown were obtained for each network from repeated network training on real data (“-“) as well as control inputs (“+”). (Right) Comparison of node weights (Pearson correlation of node weights) between different network and input types. **(c)** Similarity (Jaccard coefficient) of KPNN interpretations (differential node weights) of all pairs of control networks and the TCR KPNN. **(d-f)** Enrichment of known TCR signaling regulators among the nodes prioritized by the TCR KPNN and the control networks based on raw node weights (panel d), control node weights (panel e), and differential node weights (panel f). Enrichment is retained in control networks trained on raw data (panel d) or on control inputs (panel e), likely due to the data-independent influences of network structure on node weights. In contrast, the normalized values (differential node weights) resulted in a loss of enrichment in control networks (panel f). Enrichment scores for the controls networks are shown as a cumulative distribution, with the enrichment score for the TCR KPNN indicated by a blue line. **(g)** Control networks that are more similar to the TCR KPNN (Jaccard coefficient) show higher enrichment of known TCR signaling regulators.

**
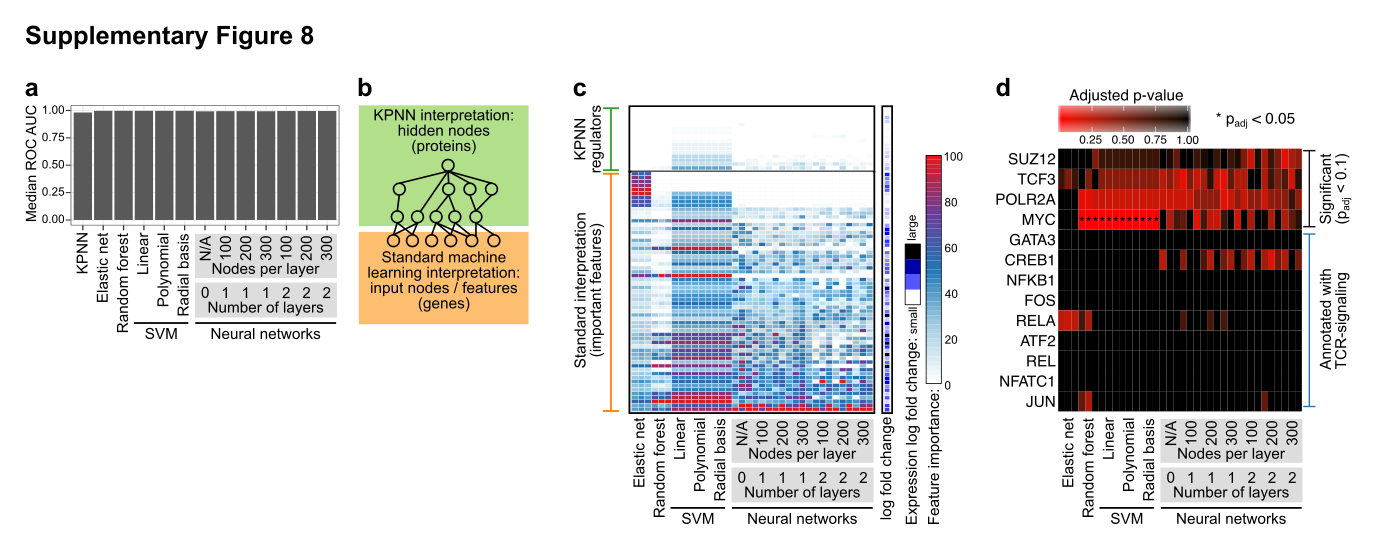
**

**Fig. S10 |** Benchmarking of KPNNs against other machine learning methods. (**a**) Prediction performance as measured by median ROC AUC values. (**b**) Conceptual comparison between KPNN interpretability, which is based on hidden nodes that reflect regulatory importance (top, green), and *ex post* interpretation of feature weights in other machine learning methods, which reflect the predictiveness of gene expression levels (bottom, orange). (**c**) Feature weights extracted from the trained machine learning methods for genes identified as important by the machine learning methods (bottom) or by the TCR KPNN (top). (**d**) Enrichment of transcription factor target genes among the 500 most important features (input nodes) of each model (analysis of the 100 or 200 most important features did not result in significant enrichments). All transcription factors with moderate significance (p_adj_ < 0.1: SUZ12, TCF3, POLR2A, and MYC), and transcription factors annotated as TCR signaling mediators (GATA3, CREB1, NFKB1, FOS, RELA, ATF2, REL, NFATC1, and JUN) are displayed. Enrichments with strong significance (p_adj_ < 0.05) are marked with an asterisk.

**
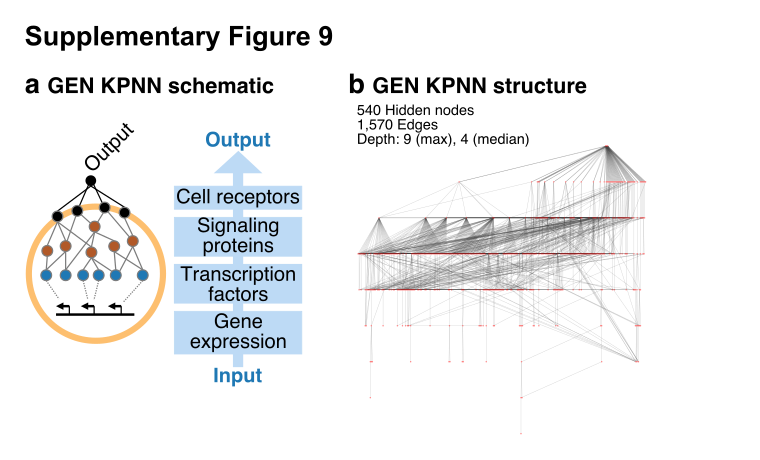
**

**Fig. S11** **|** Network structure of the GEN KPNN. (**a**) Schematic outline of the GEN KPNN. Based on public databases of signaling pathways and gene-regulatory interactions, the cell surface receptors are linked via signaling pathways to transcription factors, which are further linked to their target genes. Gene expression data provide the input to the learning algorithm. In the GEN KPNN, output specific to the biological application is predicted from all annotated cell surface receptors. (**b**) Structure of the GEN KPNN, which predicts cell states from single-cell gene expression profiles.

**
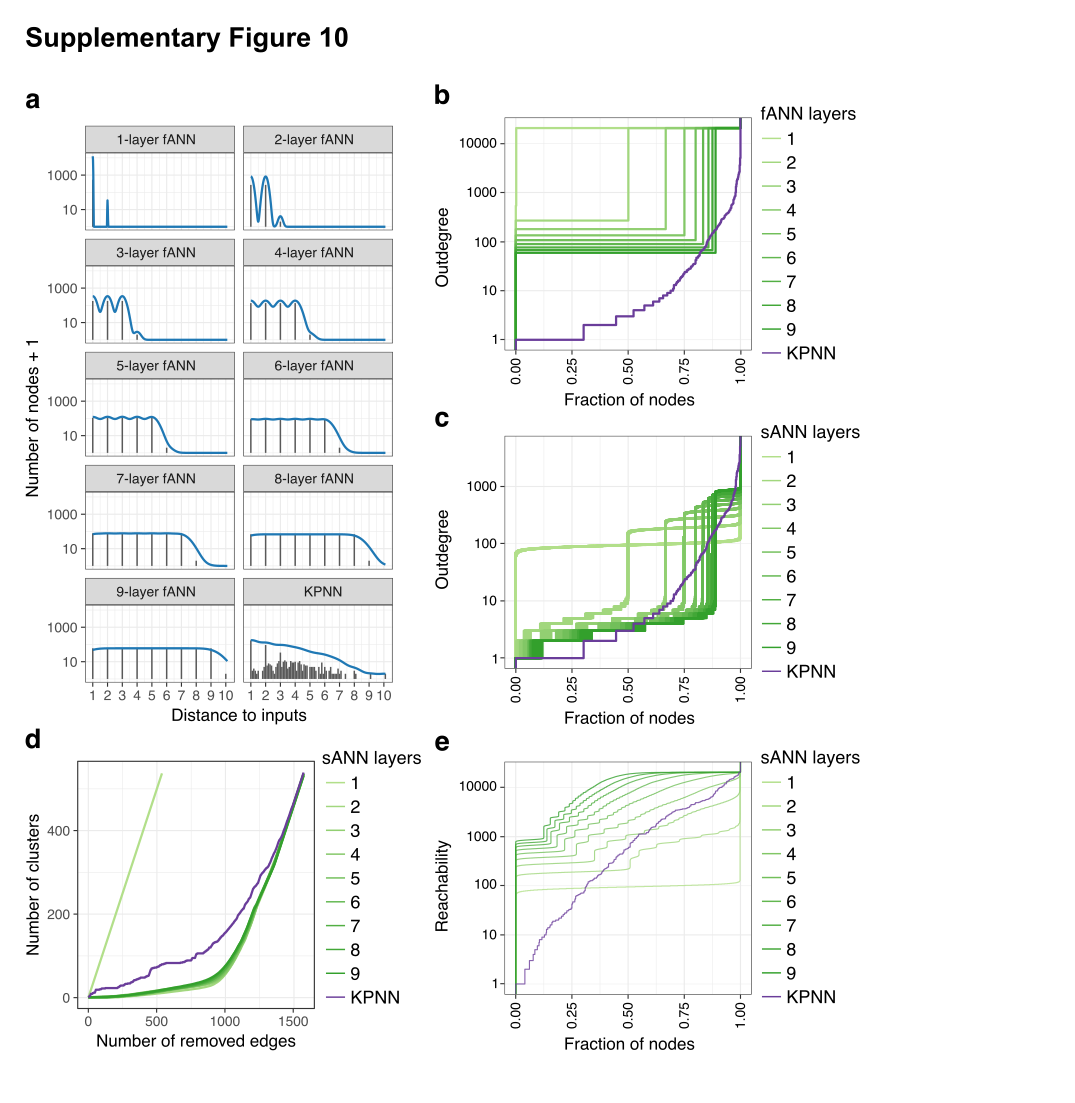
**

**Fig. S12 I** Comparison of network structure between the GEN KPNN and corresponding ANNs. The GEN KPNN is compared to fully connected ANNs (fANNs) with the same number of nodes as the KPNN, and with sparse ANNs (sANNs, n = 50 networks) in which edges were randomly removed to mimic the sparsity of the GEN KPNN. **(a)** Distribution of network distances (average distance of each hidden node to all input nodes) in the KPNN and in the fANNs. **(b)** Cumulative distribution of outdegree (number of downstream neighbors of each node) of hidden nodes in KPNN and in the fANNs. **(c)** Same as panel b, but comparing KPNNs to sANNs instead of fANNs. **(d)** Network sensitivity to targeted edge removal in the KPNN and the sANNs. Edges were removed based on their betweenness centrality values, and the number of disconnected clusters is plotted after removal of each edge. **(e)** Cumulative distribution of reachability of hidden nodes in the KPNN and in the sANNs. Reachability values measure the number of input nodes that a hidden node can access directly or indirectly (i.e., the number of inputs that are available to the hidden node).

**
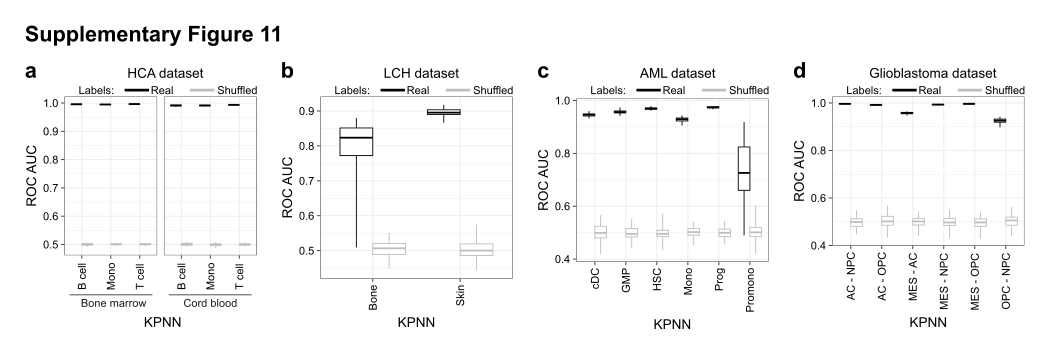
**

**Fig. S13 |** Prediction performance of KPNNs trained on four single-cell RNA-seq datasets. ROC AUC values are shown for the classification of: **(a)** Immune cell types from the Human Cell Atlas (HCA), **(b)** progenitor-like vs. mature cells for Langerhans cell histiocytosis (LCH), **(c)** leukemic vs normal cells for acute myeloid leukemia (AML), and **(d)** molecularly defined disease subtypes for glioblastoma.
